# Supplementary material for: Climate variables are not the dominant predictor of Arctic shorebird distributions
Source: PLoS One. 2023 May 17;18(5):e0285115. doi: 10.1371/journal.pone.0285115 (PMC10191349; doi:10.1371/journal.pone.0285115)
Supplement: S1 Fig — Reprinted from ArcGIS under a CC BY license, with permission from Esri, original Copyright 2022 Esri (Basemaps supported by Esri, HERE, Garmin, OpenStreetMap contributors, and the GIS User Community). (PDF) [file pone.0285115.s001.pdf]

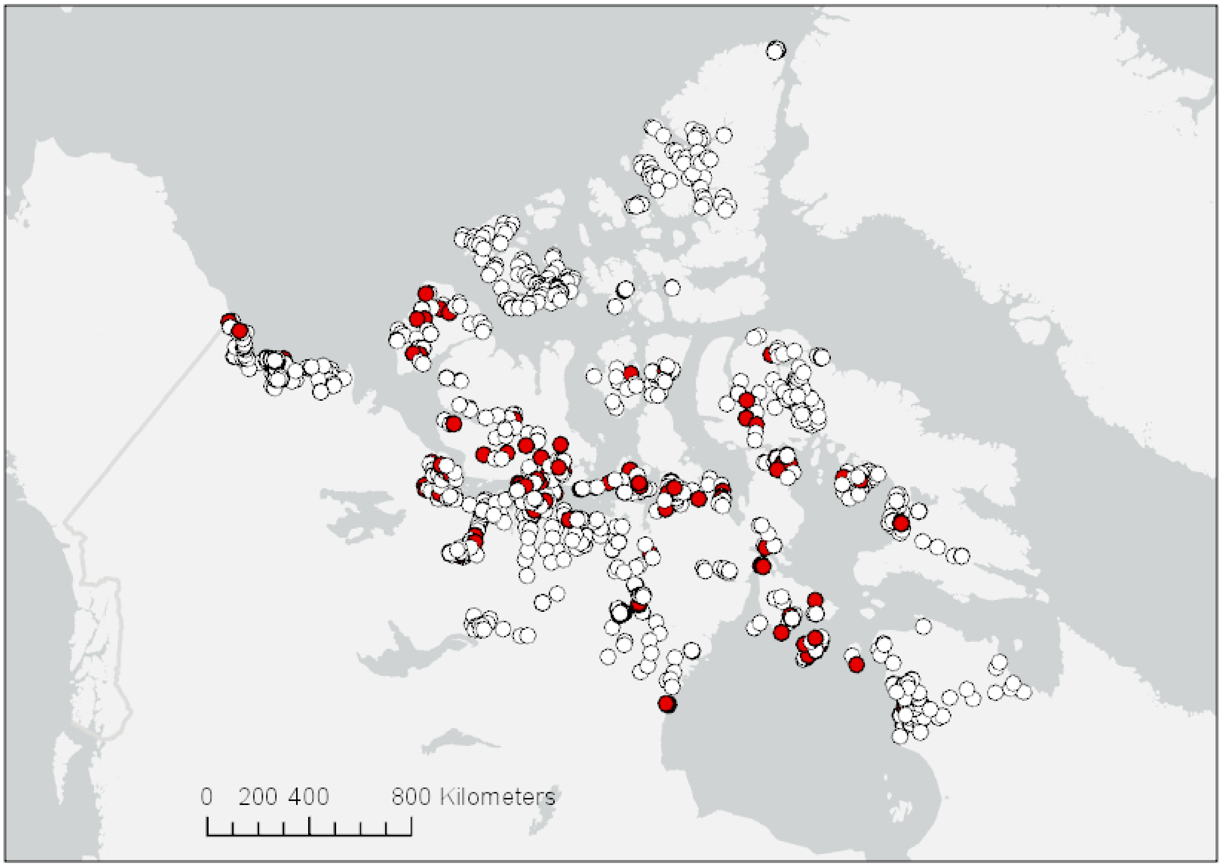

American Golden-plover

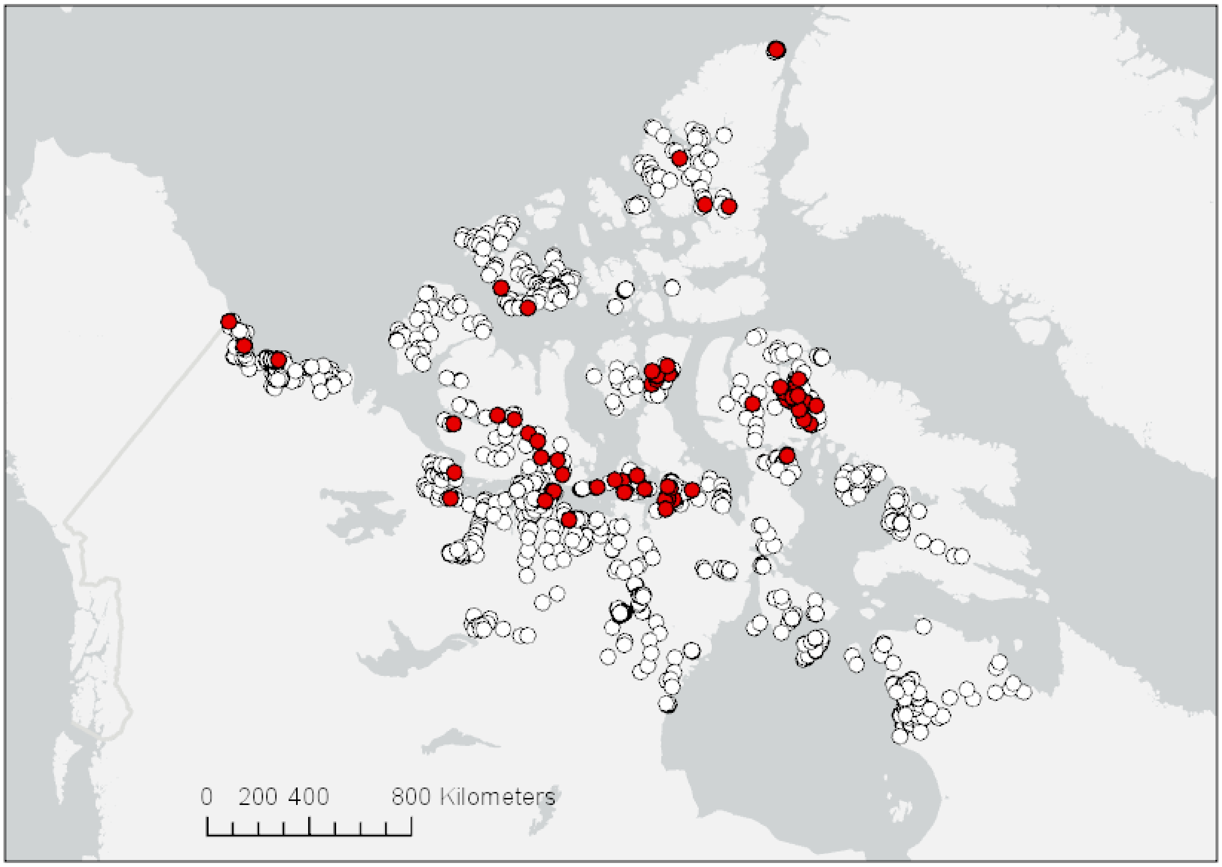

Baird’s Sandpiper

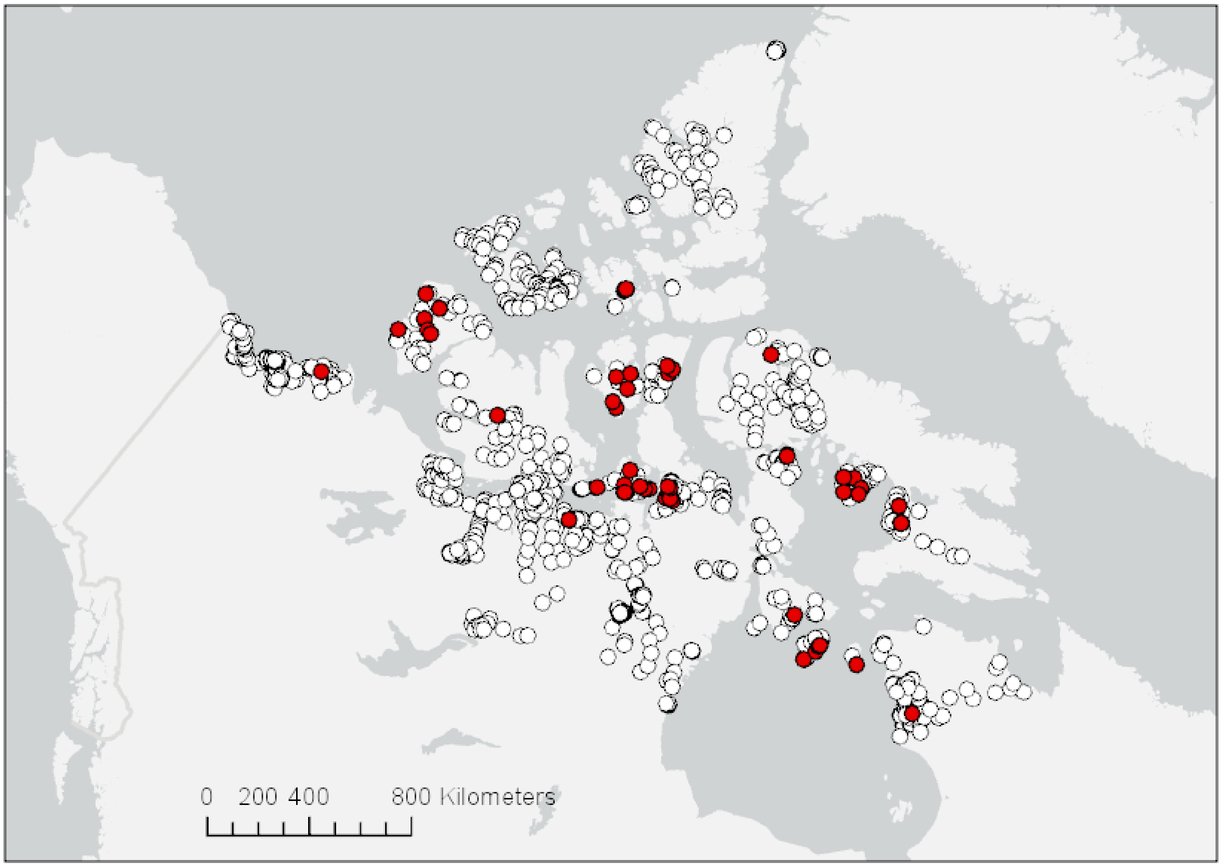

Black-bellied Plover

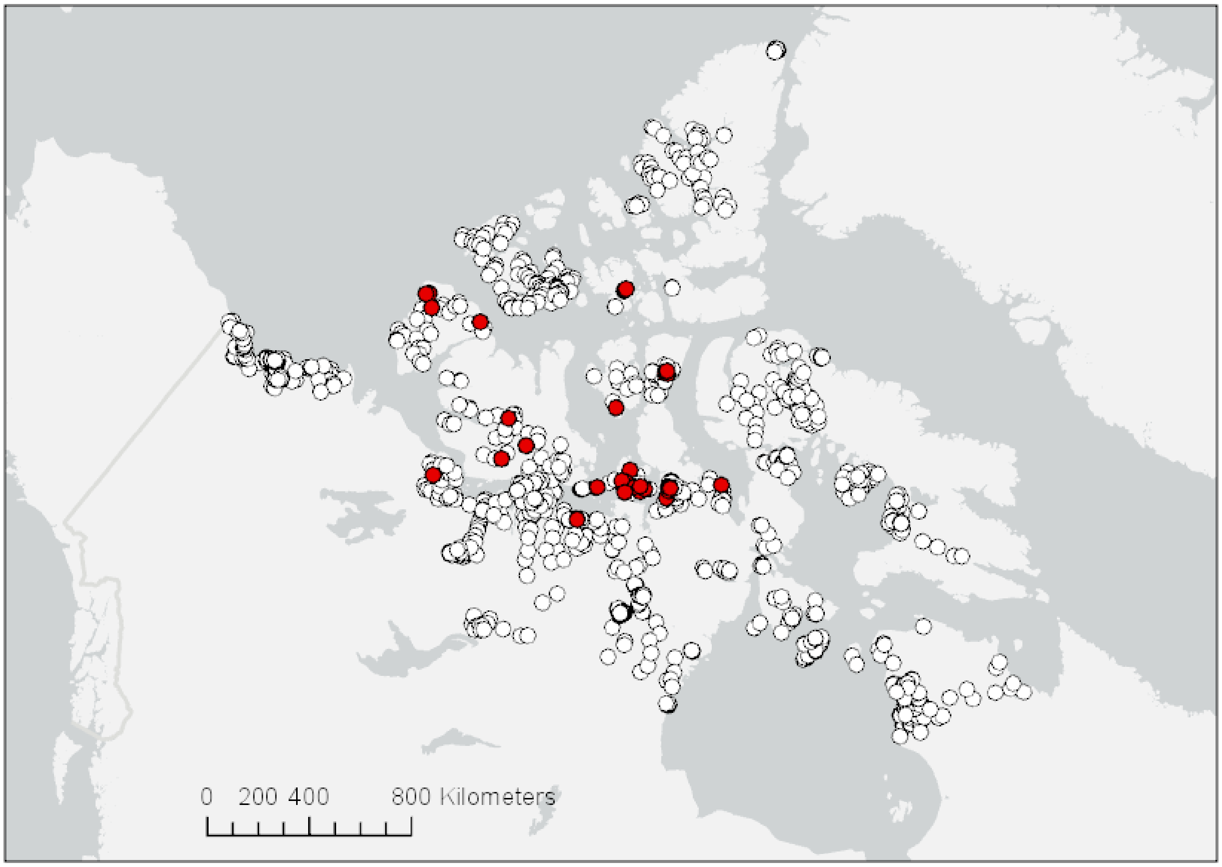

Buff-breasted Sandpiper

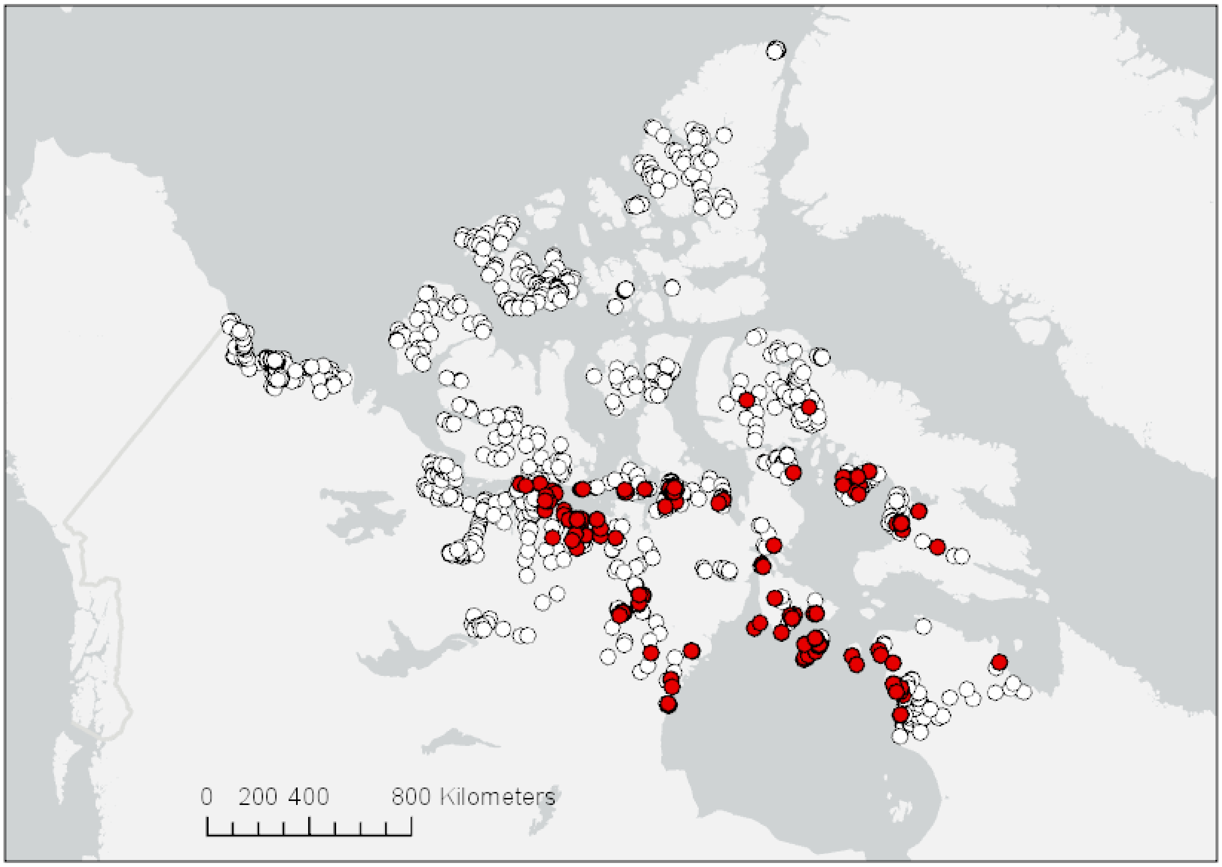

Dunlin

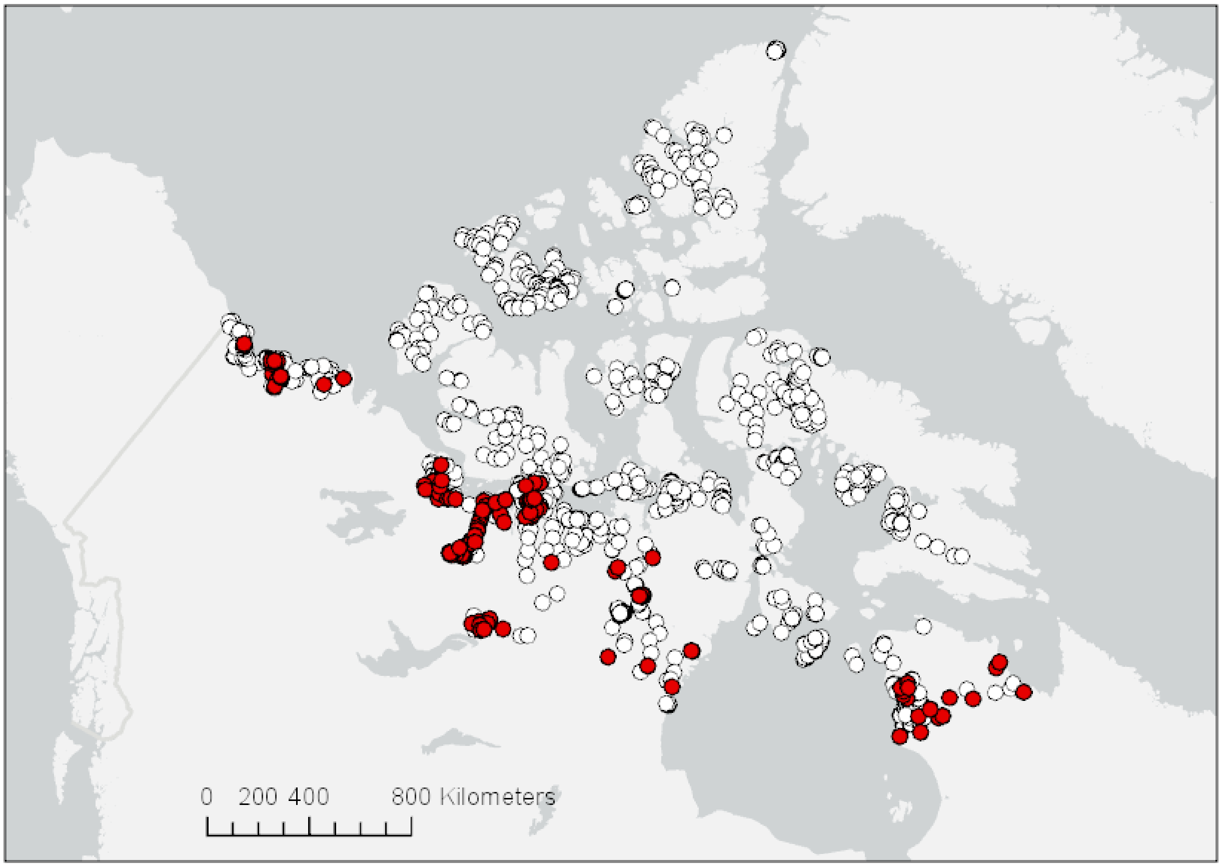

Least Sandpiper

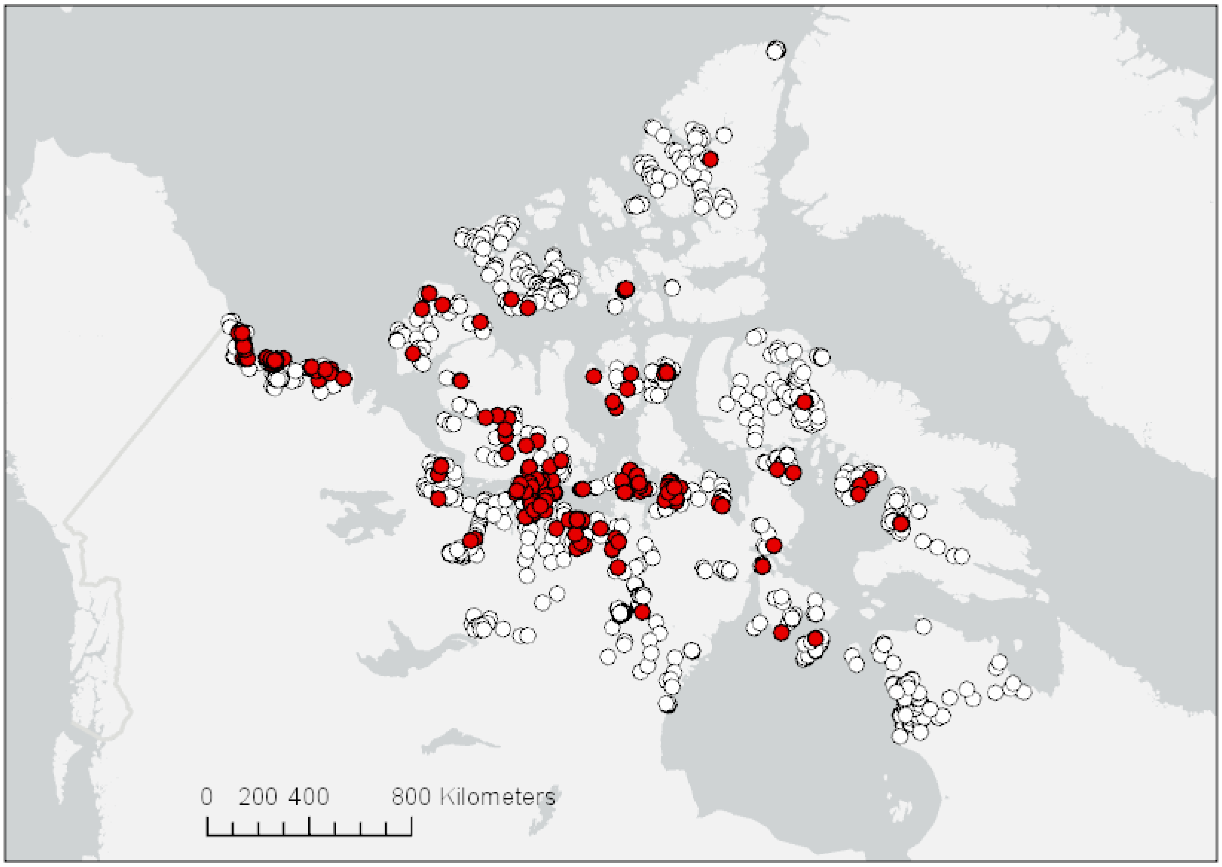

Pectoral Sandpiper

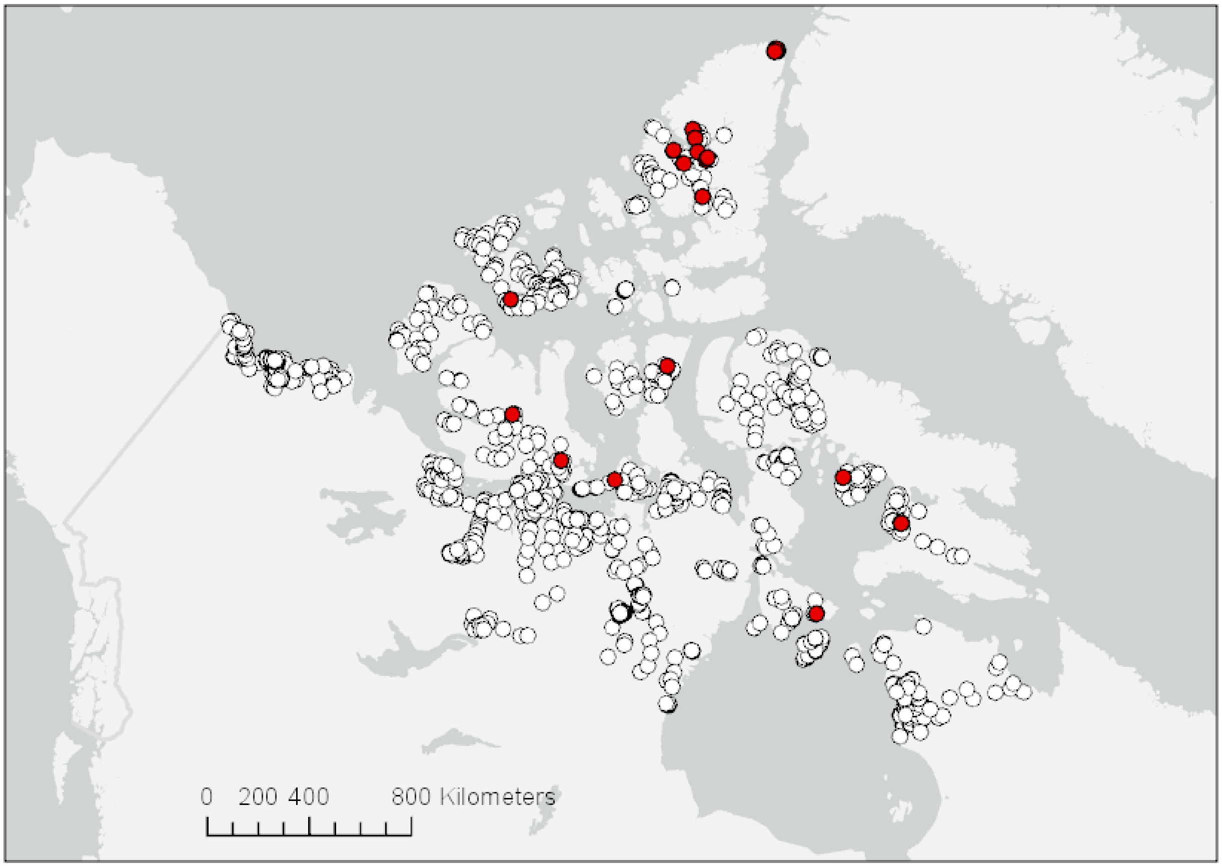

Red Knot

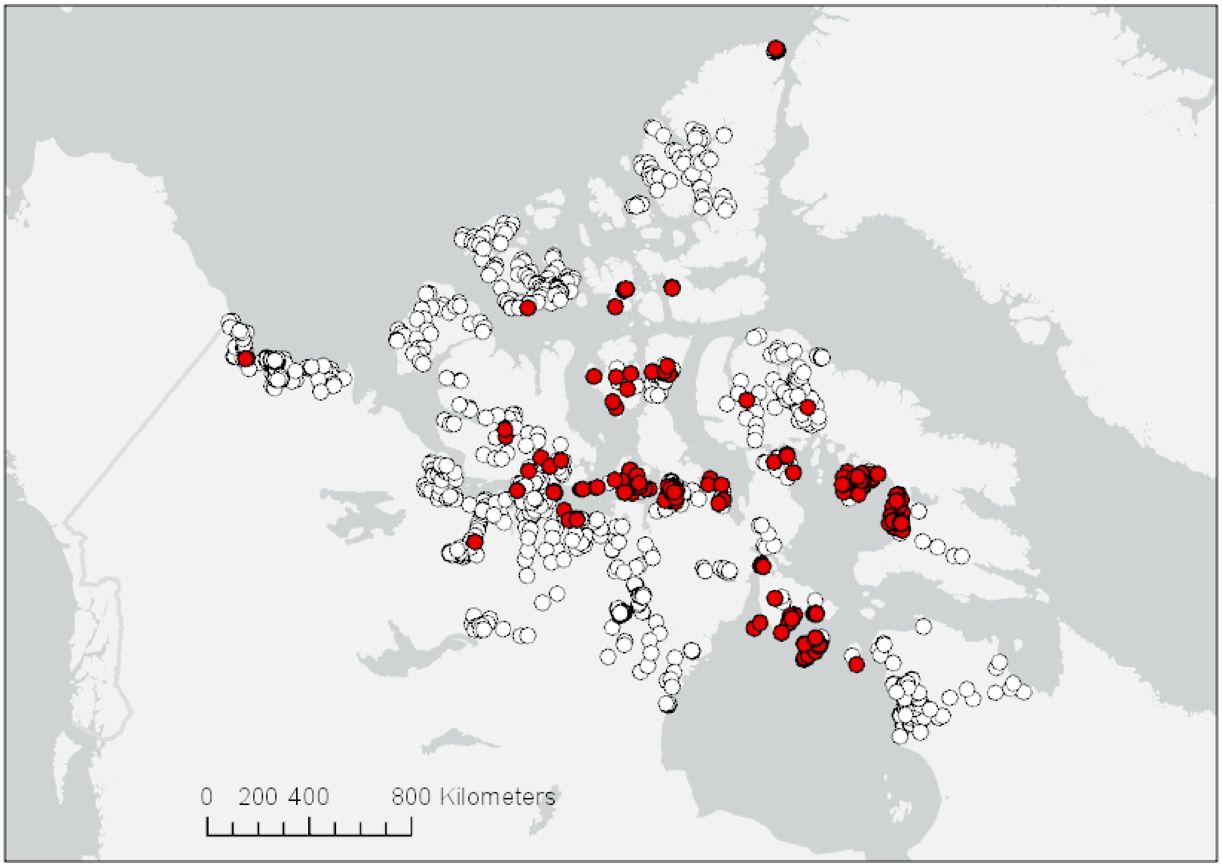

Red Phalarope

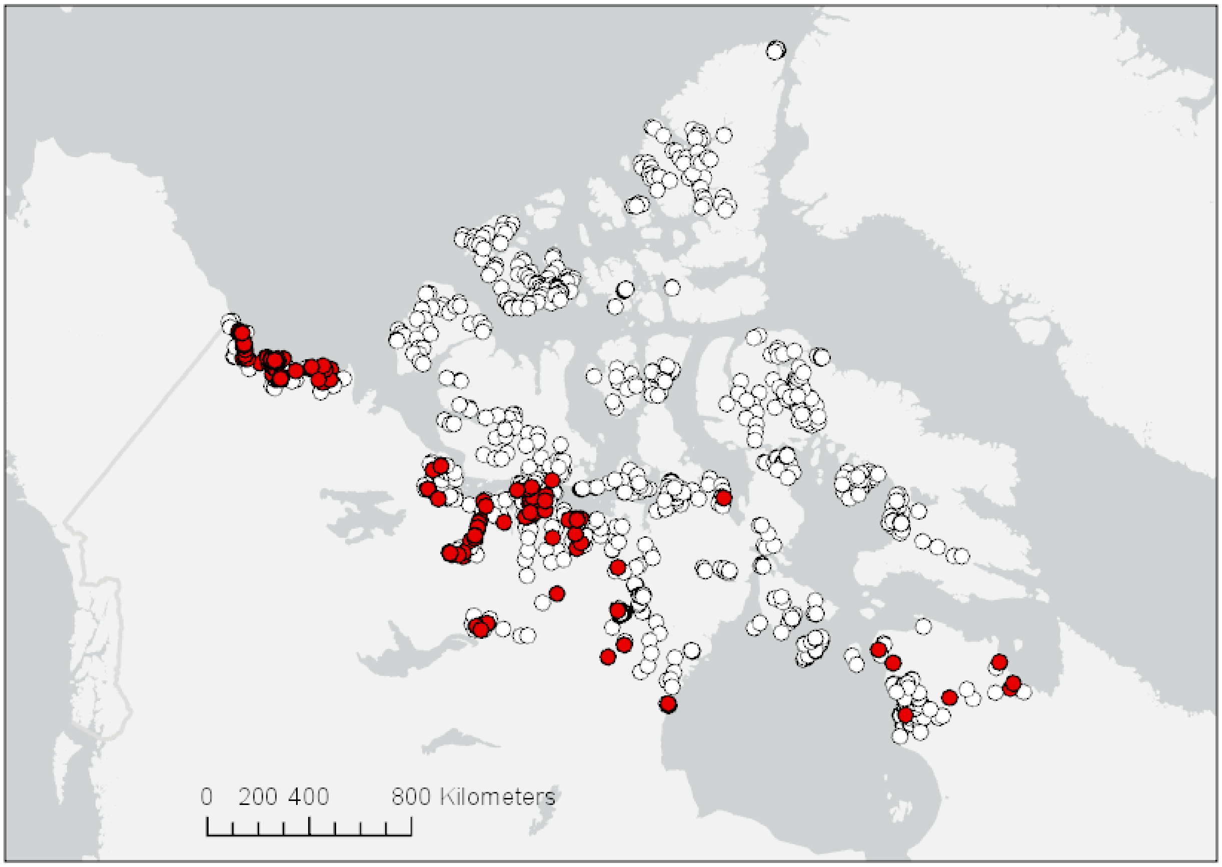

Red-necked Phalarope

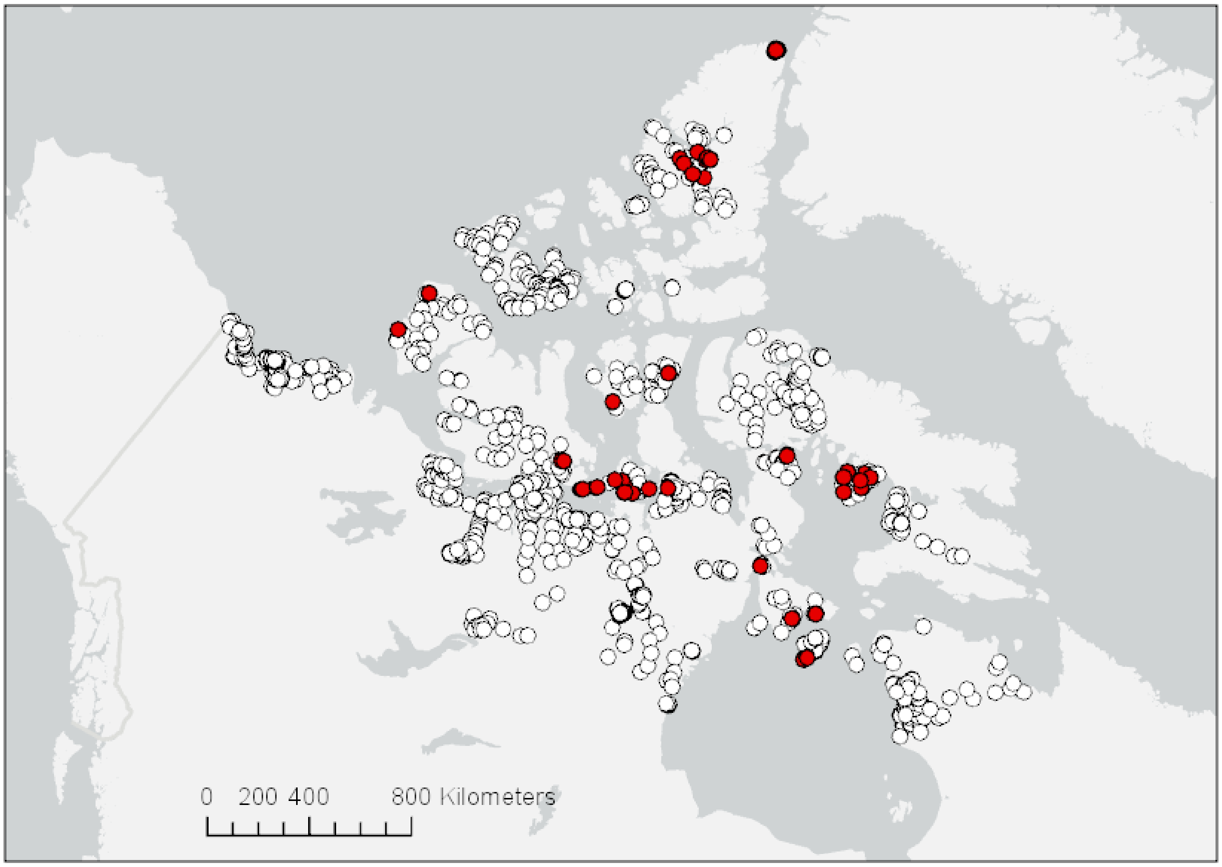

Ruddy Turnstone

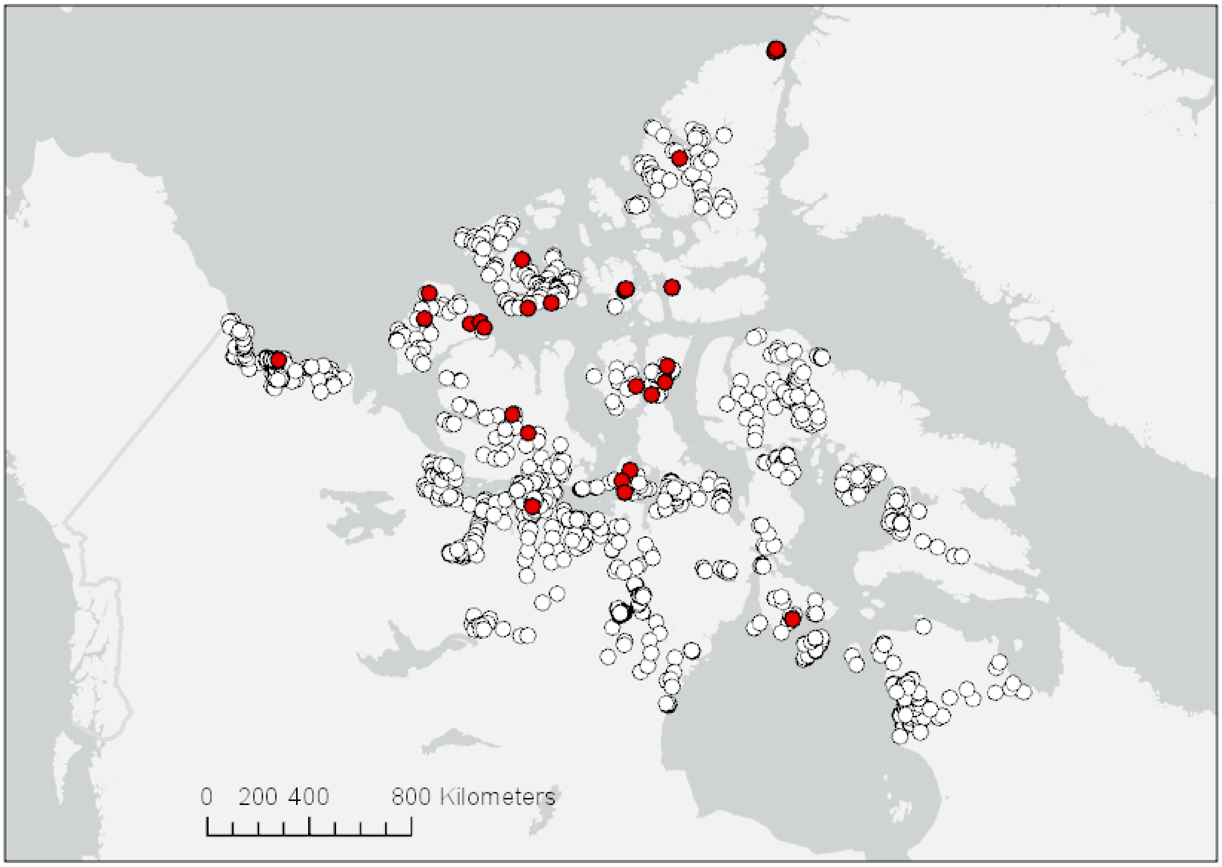

Sanderling

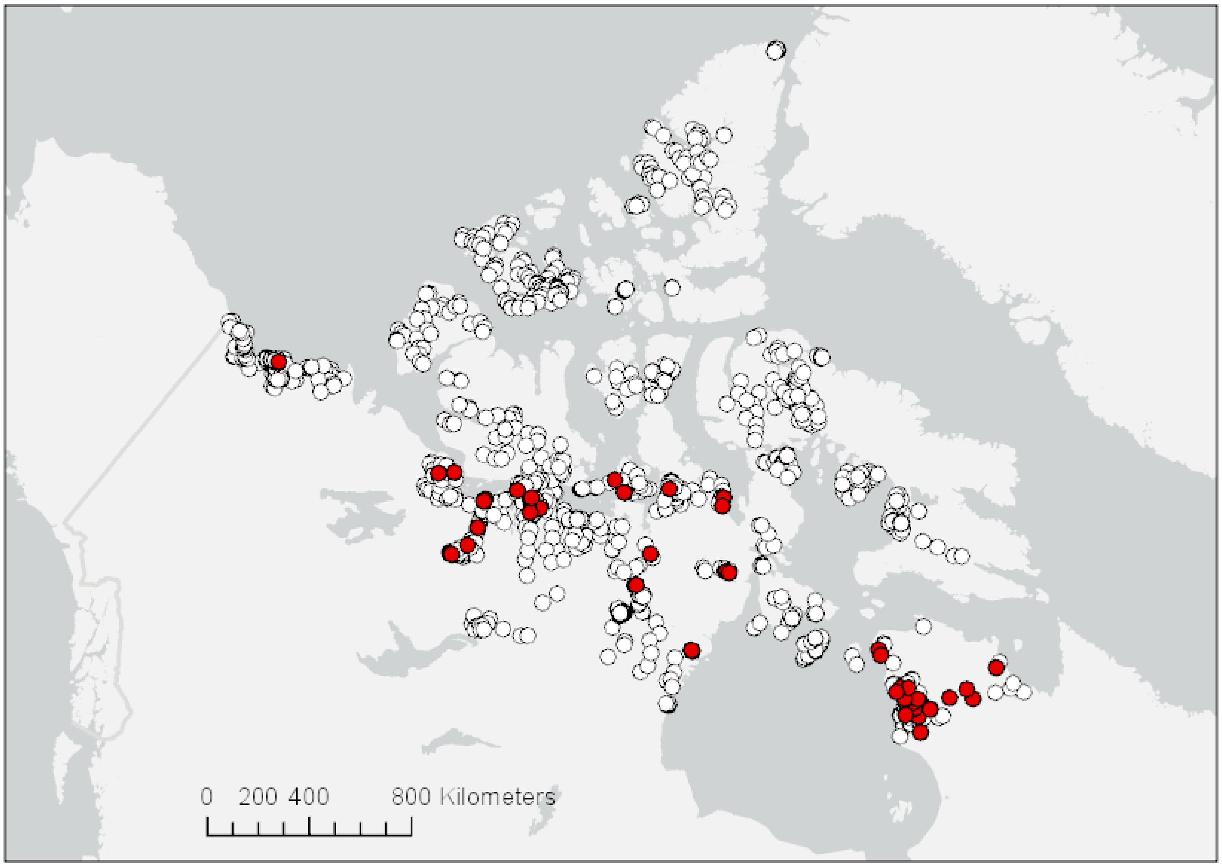

Semipalmated Plover

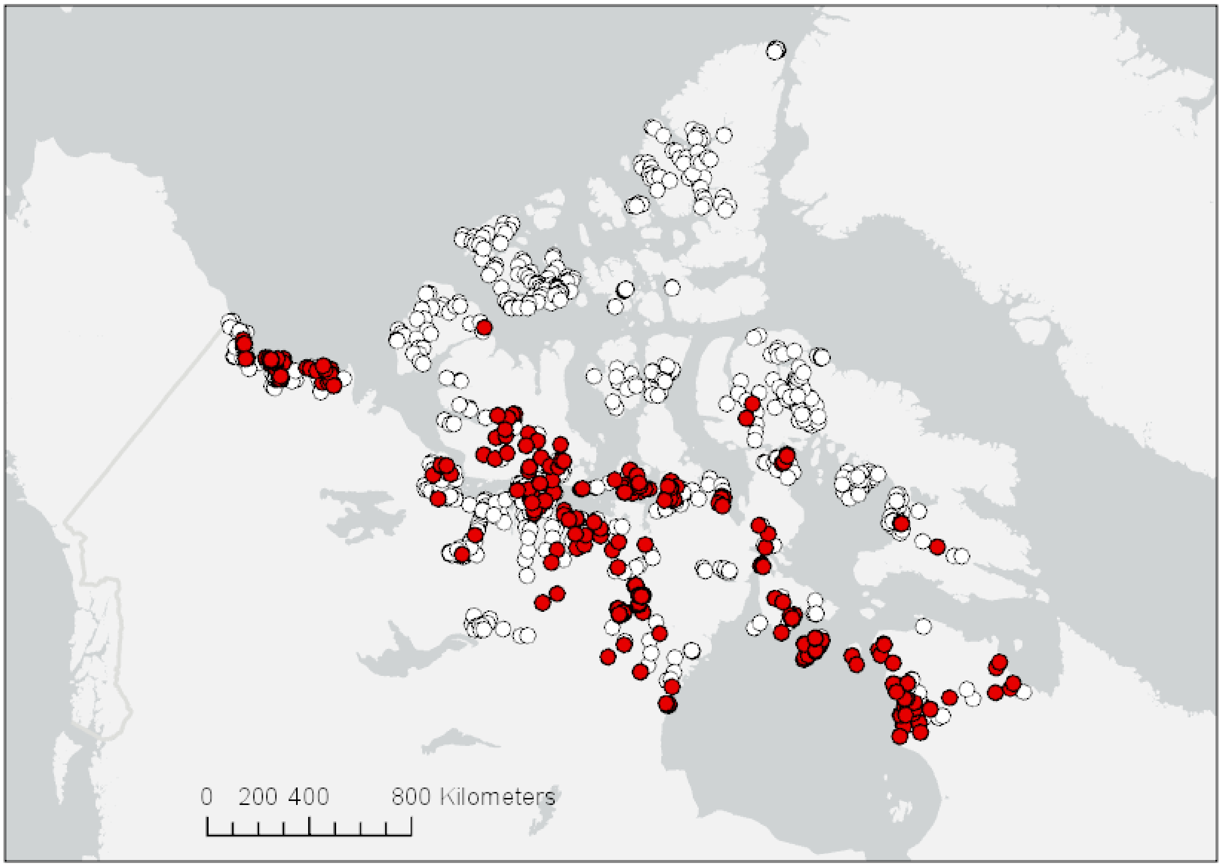

Semipalmated Sandpiper

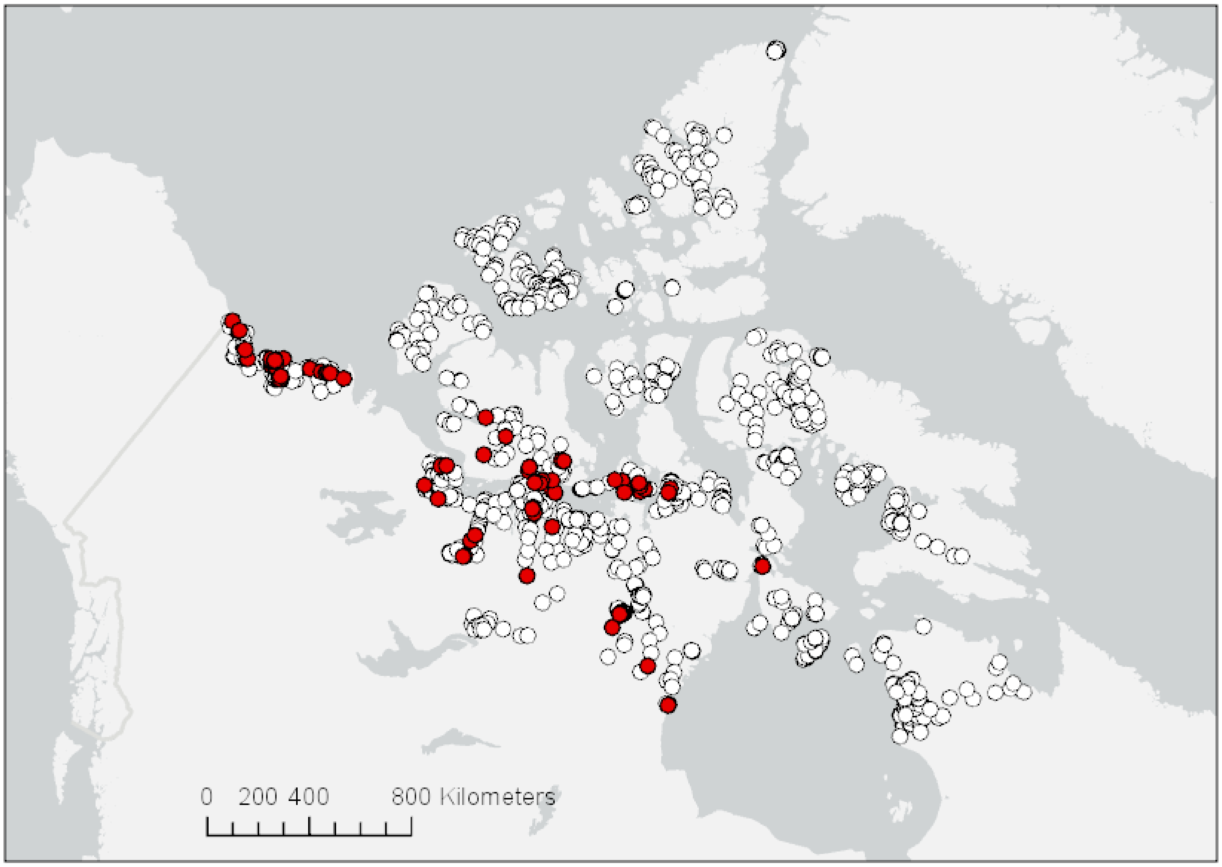

Stilt Sandpiper

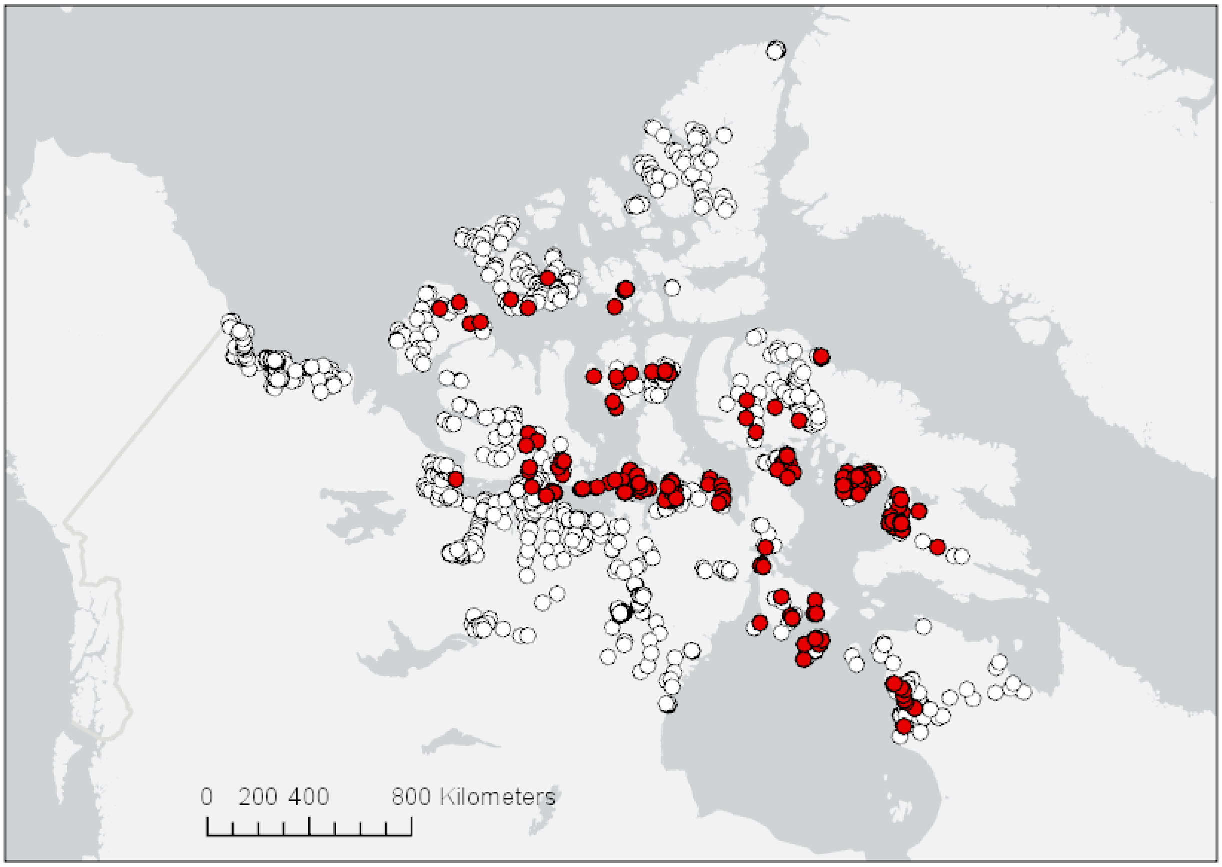

White-rumped Sandpiper

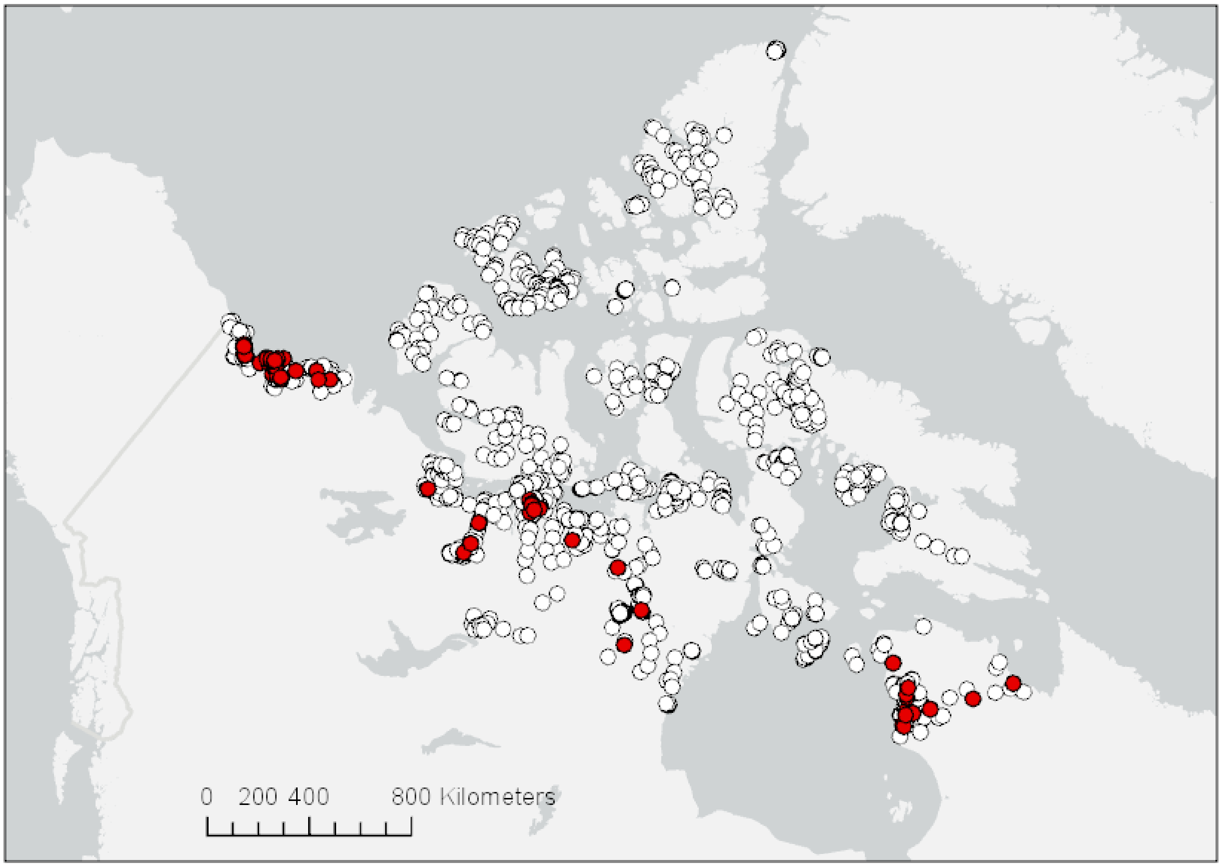

Wilson’s Snipe
