# Supplementary material for: Climate variables are not the dominant predictor of Arctic shorebird distributions
Source: PLoS One. 2023 May 17;18(5):e0285115. doi: 10.1371/journal.pone.0285115 (PMC10191349; doi:10.1371/journal.pone.0285115)
Supplement: S2 Fig — (PDF) [file pone.0285115.s002.pdf]

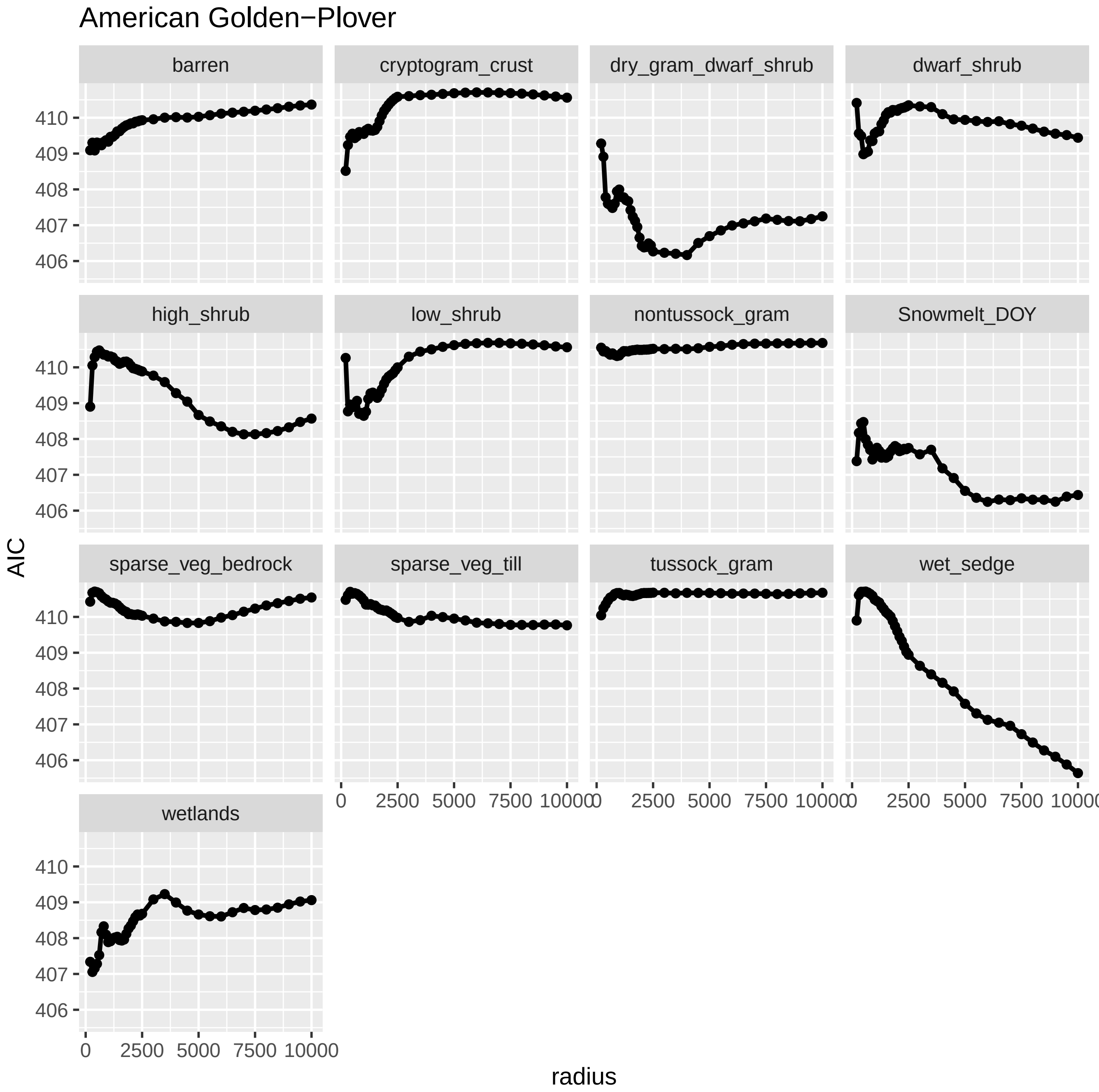
American Golden−Plover
barren
cryptogram_crust
dry_gram_dwarf_shrub
dwarf_shrub
high_shrub
low_shrub
nontussock_gram
Snowmelt_DOY
sparse_veg_bedrock
sparse_veg_till
tussock_gram
wet_sedge
wetlands
AIC
406
407
408
409
410
radius
0
2500
5000
7500
10000

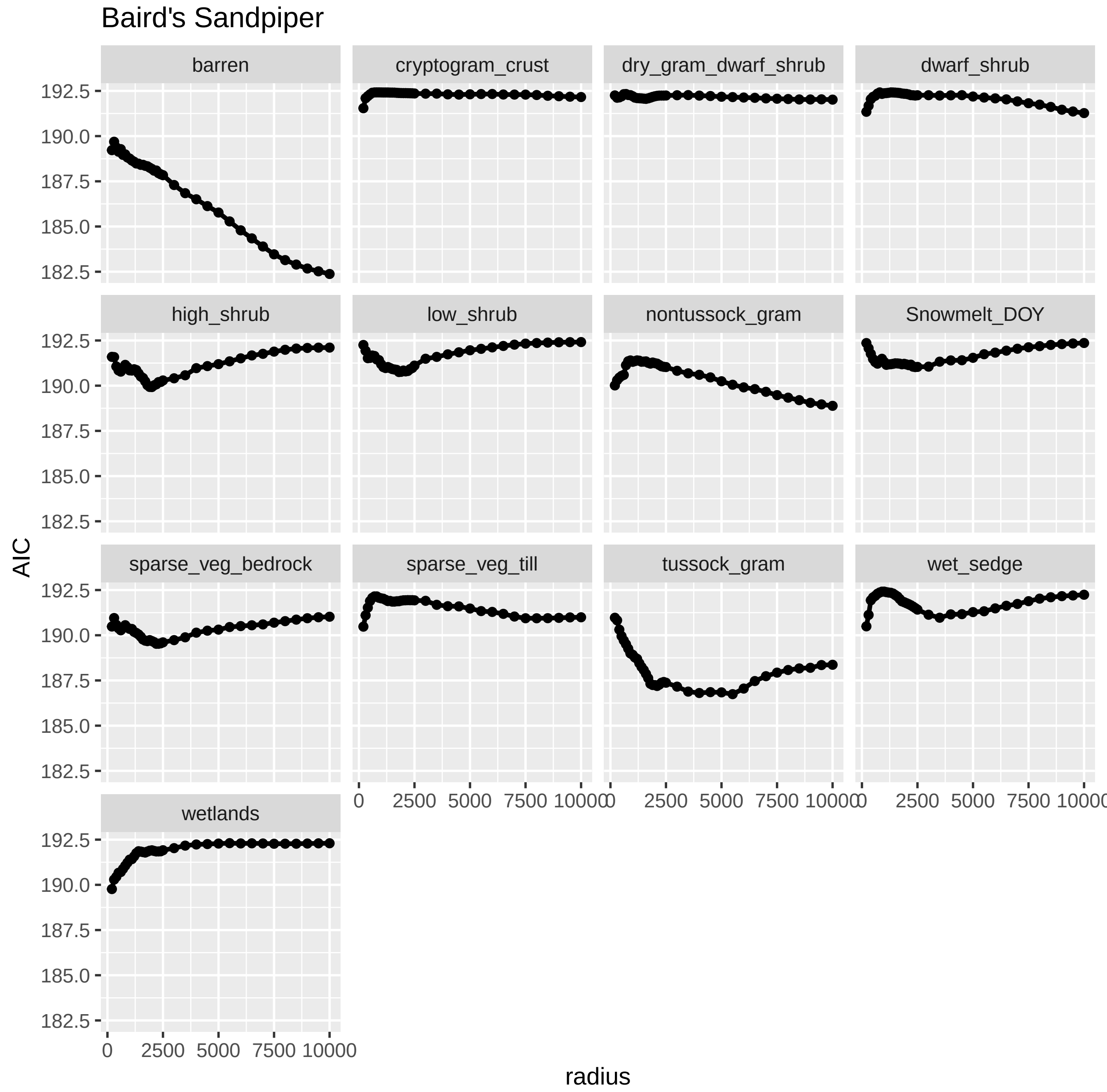

Baird's Sandpiper
barren
cryptogram_crust
dry_gram_dwarf_shrub
dwarf_shrub
high_shrub
low_shrub
nontussock_gram
Snowmelt_DOY
sparse_veg_bedrock
sparse_veg_till
tussock_gram
wet_sedge
wetlands
AIC
192.5
190.0
187.5
185.0
182.5
0
2500
5000
7500
10000
radius

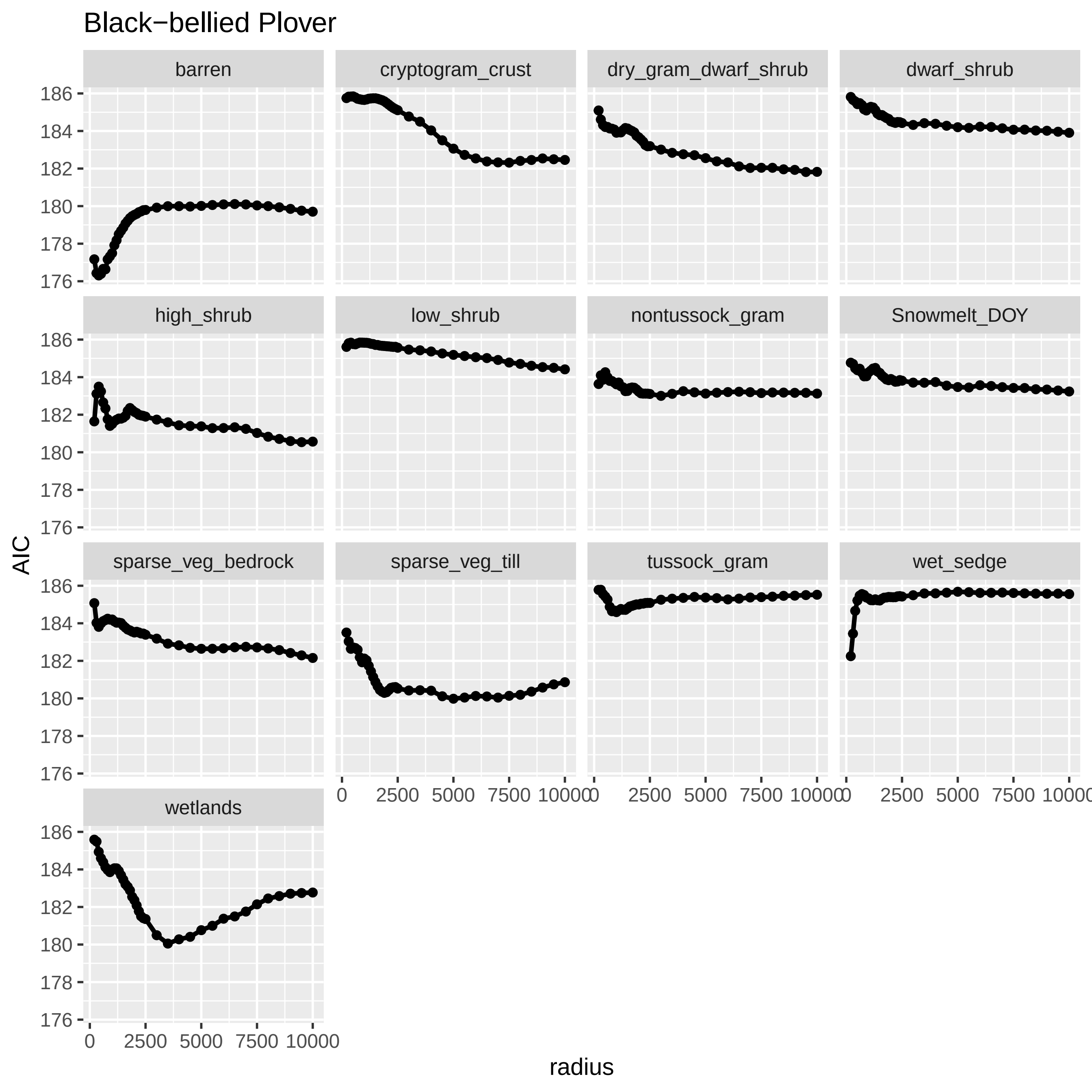

Black−bellied Plover
barren
cryptogram_crust
dry_gram_dwarf_shrub
dwarf_shrub
high_shrub
low_shrub
nontussock_gram
Snowmelt_DOY
sparse_veg_bedrock
sparse_veg_till
tussock_gram
wet_sedge
wetlands
AIC
176
178
180
182
184
186
0
2500
5000
7500
10000
radius

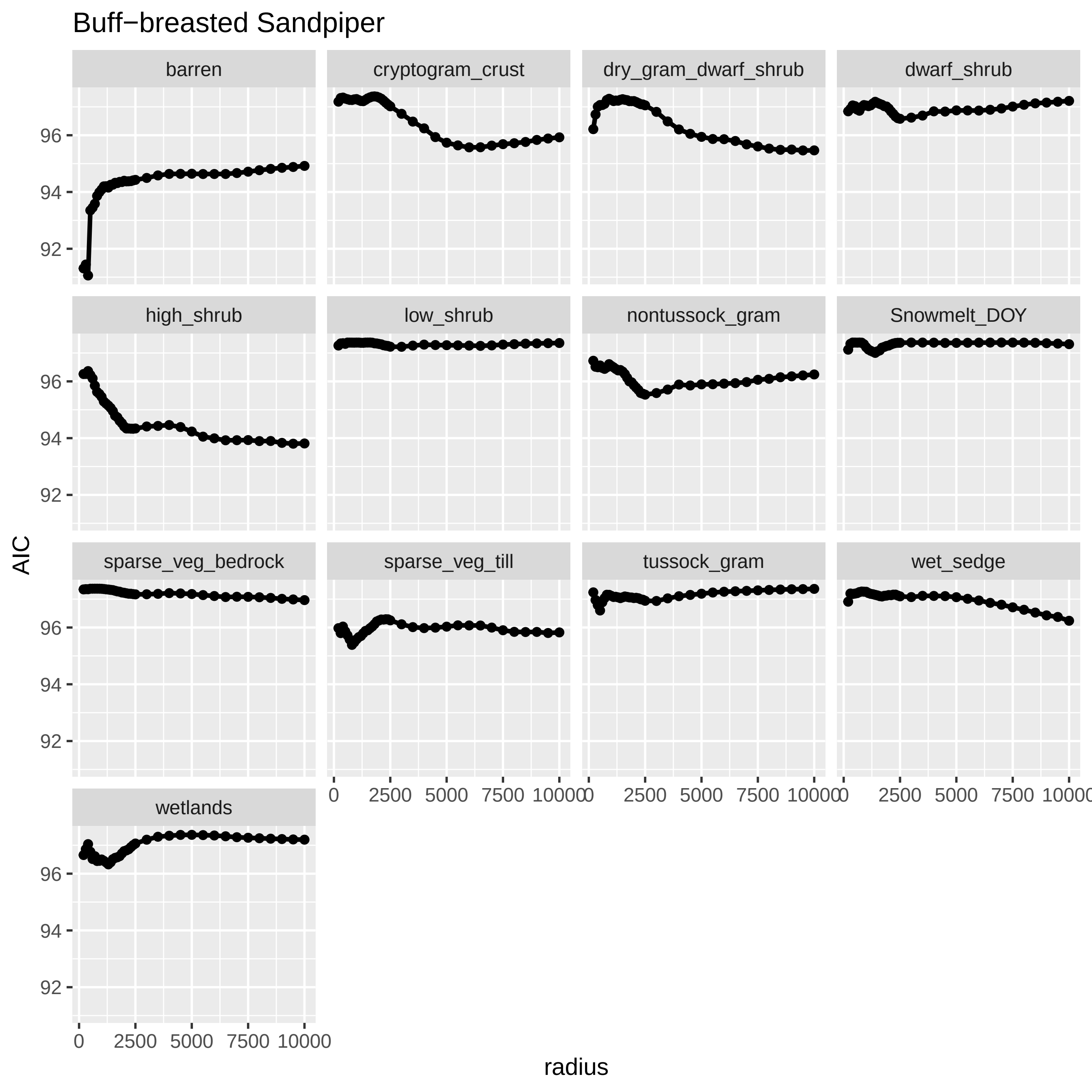
Buff−breasted Sandpiper
barren
cryptogram_crust
dry_gram_dwarf_shrub
dwarf_shrub
high_shrub
low_shrub
nontussock_gram
Snowmelt_DOY
sparse_veg_bedrock
sparse_veg_till
tussock_gram
wet_sedge
wetlands
AIC
92
94
96
radius
0
2500
5000
7500
10000

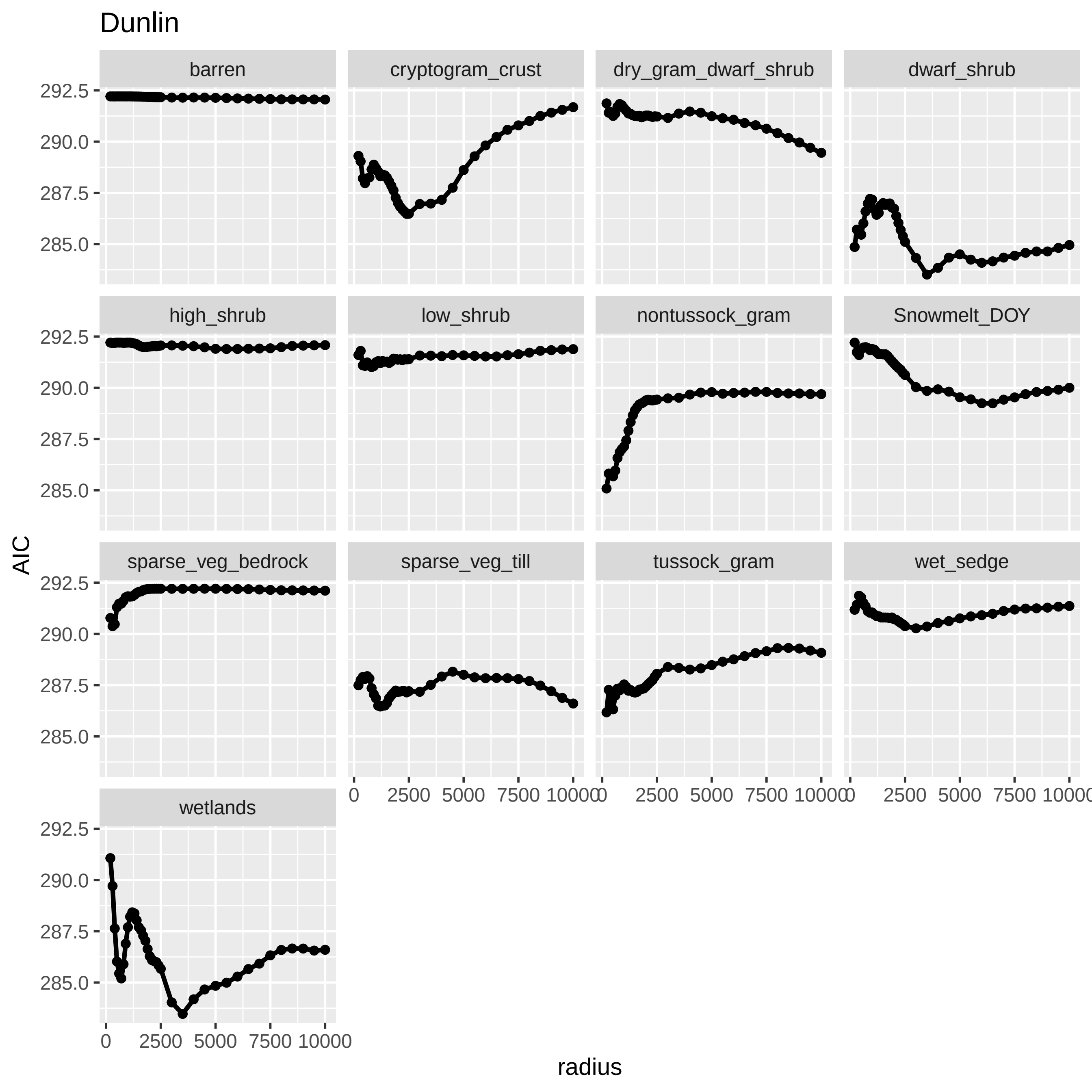
Dunlin
barren
cryptogram_crust
dry_gram_dwarf_shrub
dwarf_shrub
high_shrub
low_shrub
nontussock_gram
Snowmelt_DOY
sparse_veg_bedrock
sparse_veg_till
tussock_gram
wet_sedge
wetlands
AIC
292.5
290.0
287.5
285.0
radius
0
2500
5000
7500
10000

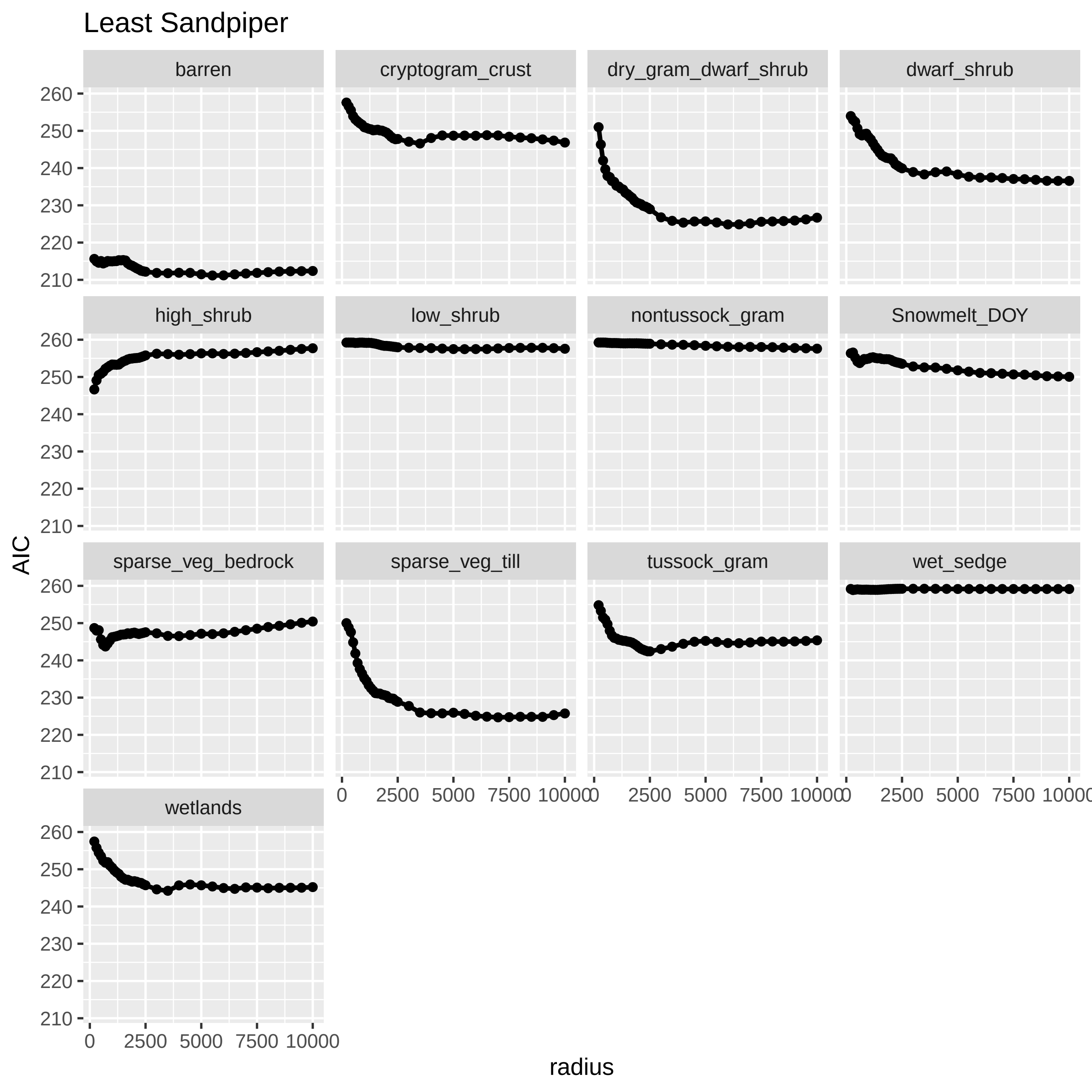
Least Sandpiper
barren
cryptogram_crust
dry_gram_dwarf_shrub
dwarf_shrub
high_shrub
low_shrub
nontussock_gram
Snowmelt_DOY
sparse_veg_bedrock
sparse_veg_till
tussock_gram
wet_sedge
wetlands
AIC
260
250
240
230
220
210
0
2500
5000
7500
10000
radius

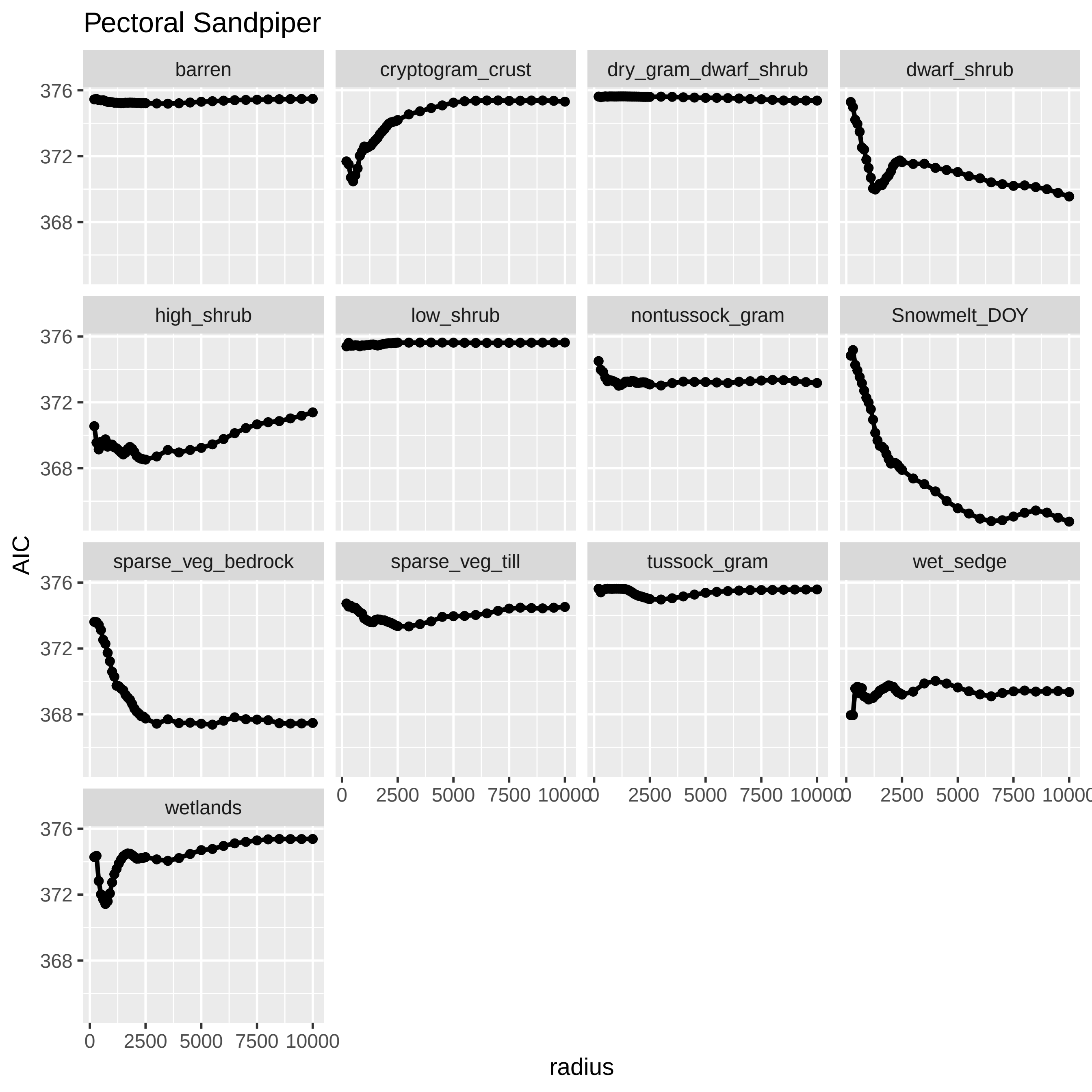
Pectoral Sandpiper
barren
cryptogram_crust
dry_gram_dwarf_shrub
dwarf_shrub
high_shrub
low_shrub
nontussock_gram
Snowmelt_DOY
sparse_veg_bedrock
sparse_veg_till
tussock_gram
wet_sedge
wetlands
AIC
376
372
368
radius
0
2500
5000
7500
10000

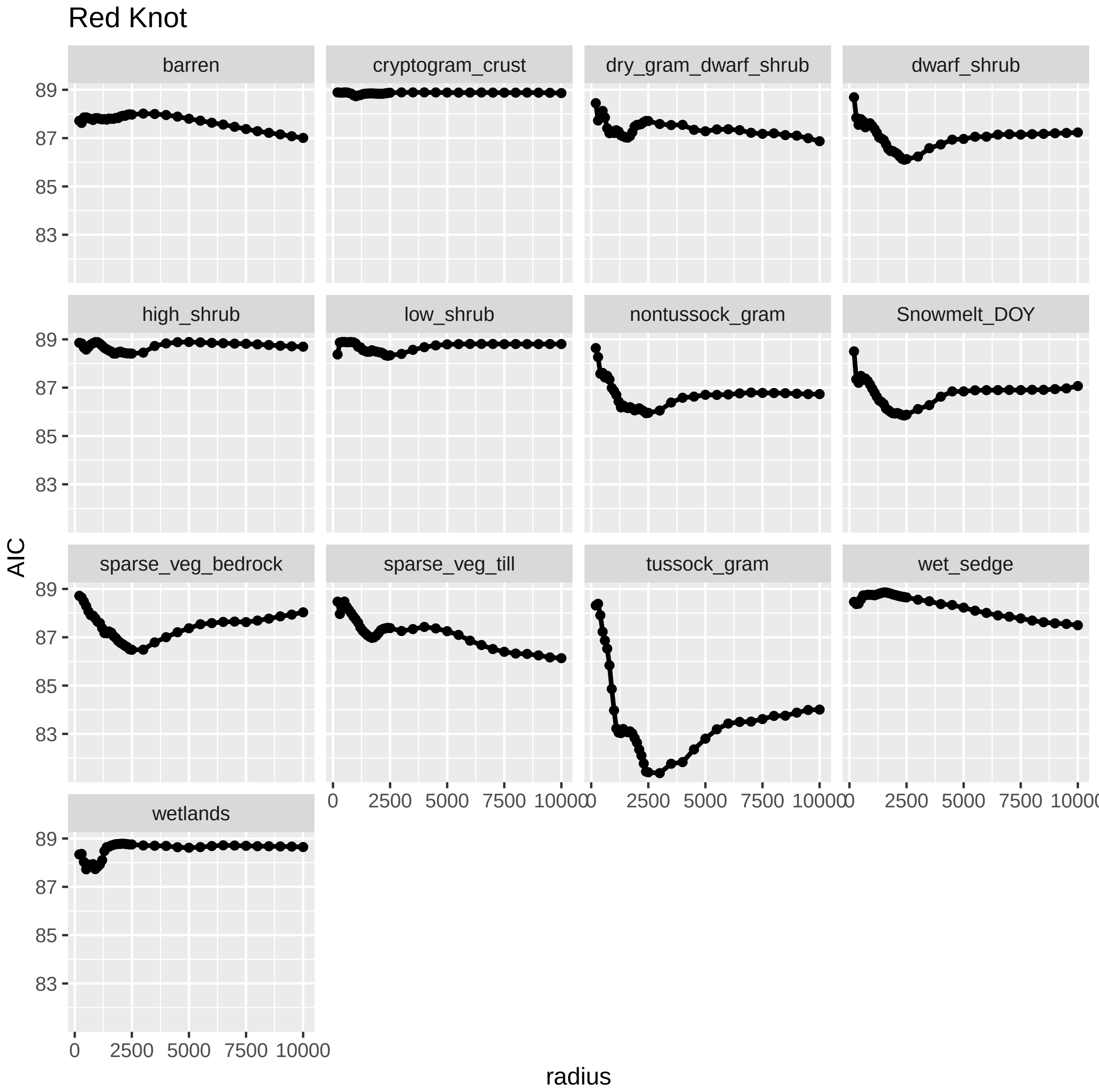

Red Knot
barren
cryptogram_crust
dry_gram_dwarf_shrub
dwarf_shrub
high_shrub
low_shrub
nontussock_gram
Snowmelt_DOY
sparse_veg_bedrock
sparse_veg_till
tussock_gram
wet_sedge
wetlands
AIC
89
87
85
83
radius
0
2500
5000
7500
10000

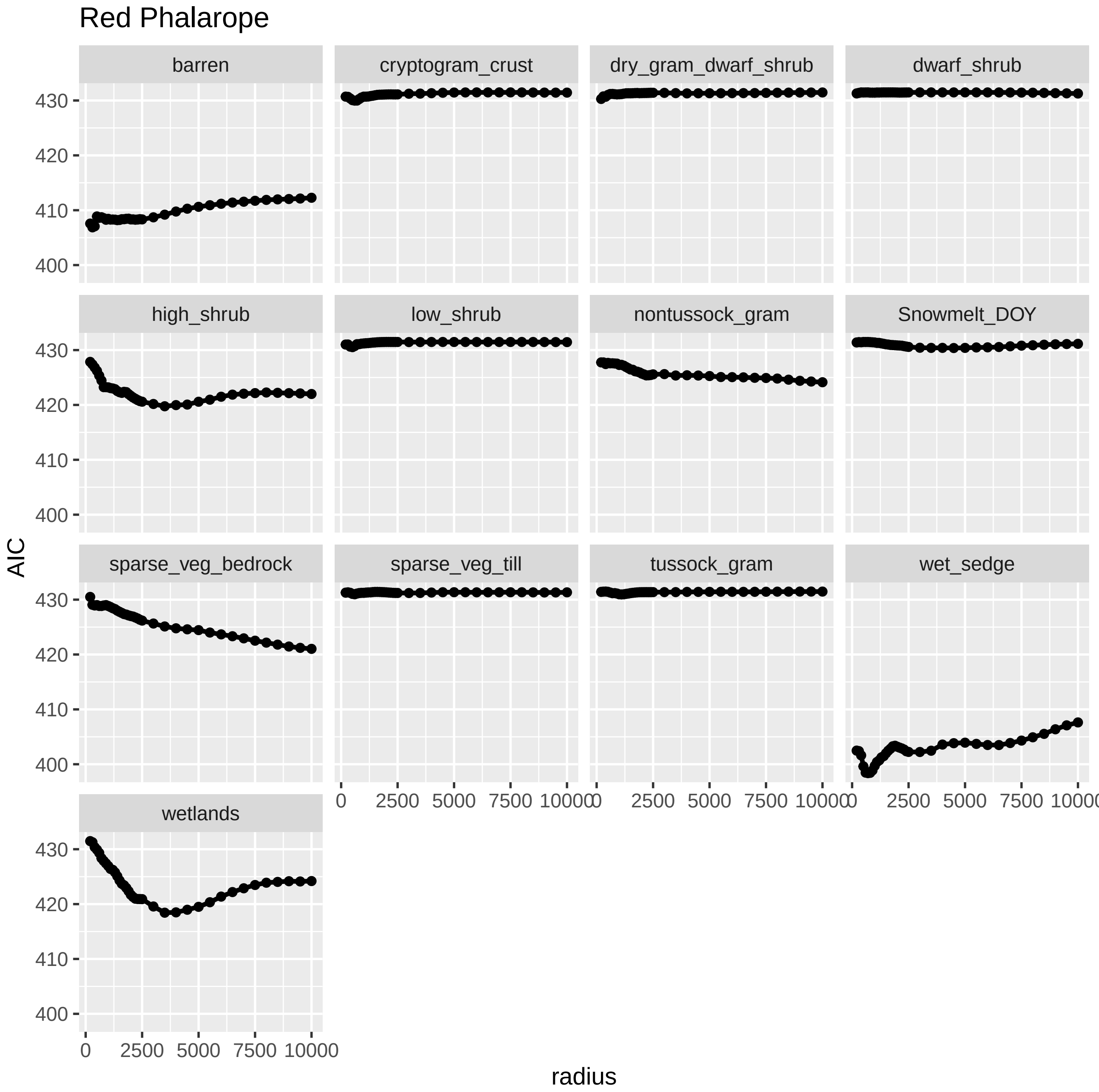
Red Phalarope
barren
cryptogram_crust
dry_gram_dwarf_shrub
dwarf_shrub
high_shrub
low_shrub
nontussock_gram
Snowmelt_DOY
sparse_veg_bedrock
sparse_veg_till
tussock_gram
wet_sedge
wetlands
AIC
radius
400
410
420
430
0
2500
5000
7500
10000

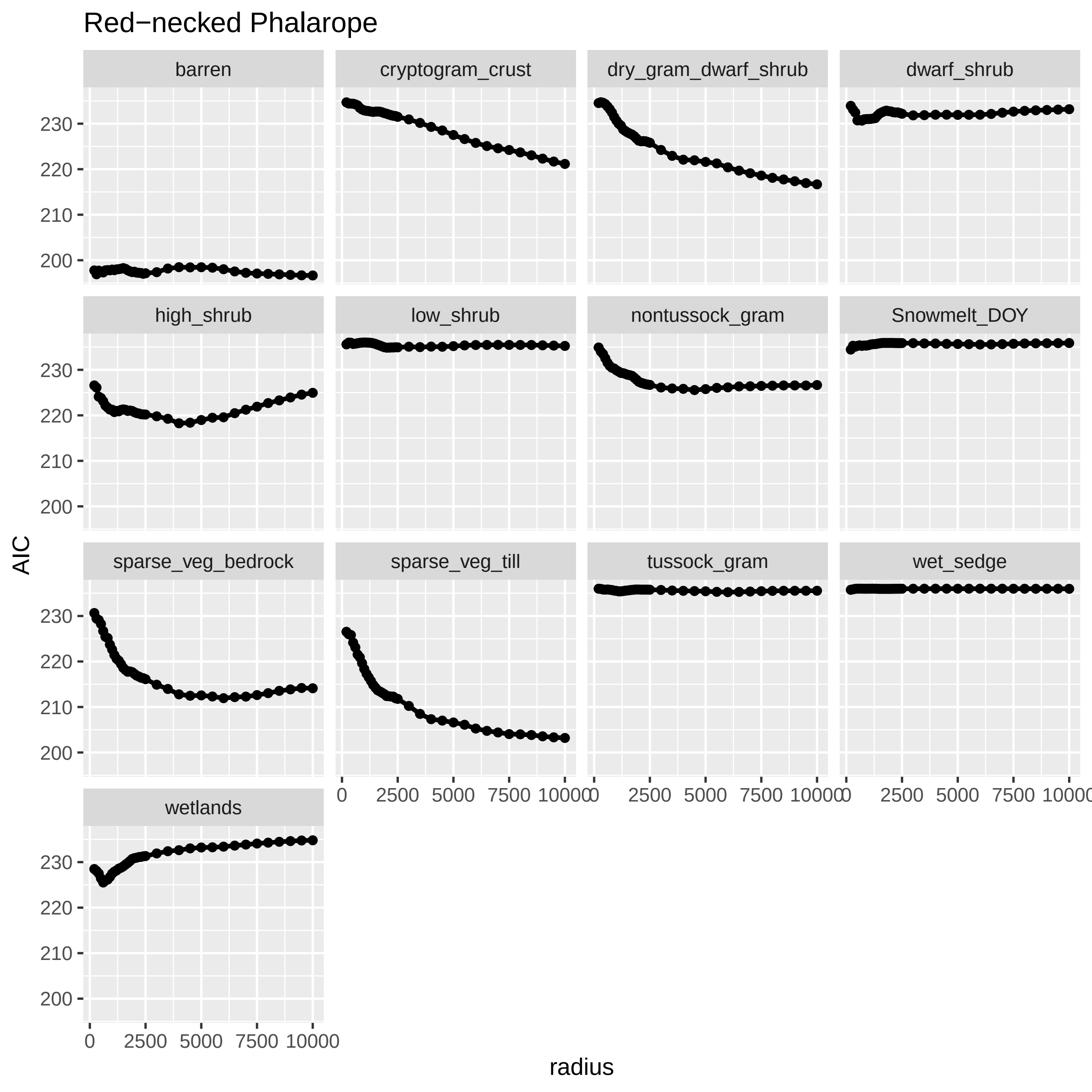
Red−necked Phalarope
barren
cryptogram_crust
dry_gram_dwarf_shrub
dwarf_shrub
high_shrub
low_shrub
nontussock_gram
Snowmelt_DOY
sparse_veg_bedrock
sparse_veg_till
tussock_gram
wet_sedge
wetlands
AIC
200
210
220
230
0
2500
5000
7500
10000
radius

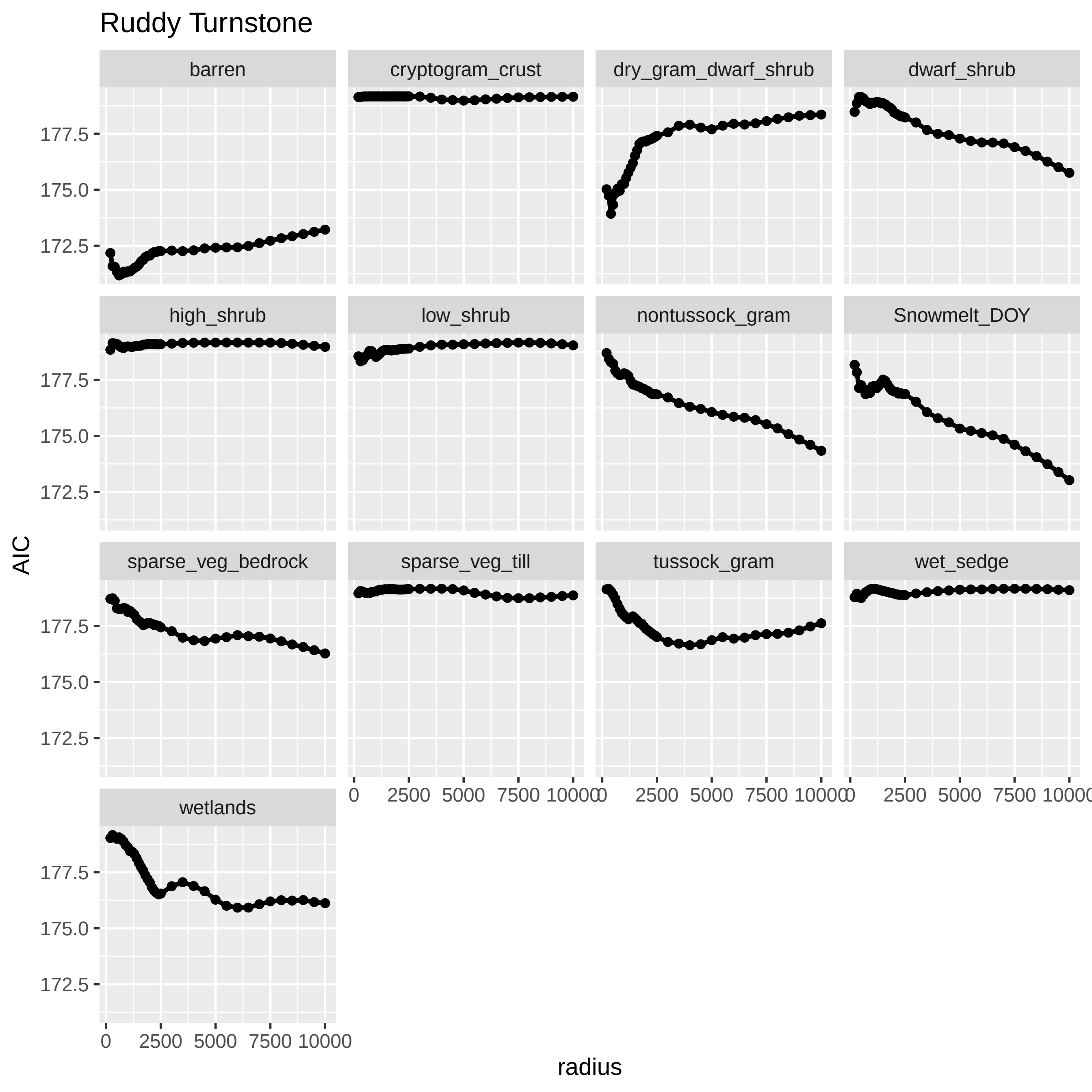
Ruddy Turnstone
barren
cryptogram_crust
dry_gram_dwarf_shrub
dwarf_shrub
high_shrub
low_shrub
nontussock_gram
Snowmelt_DOY
sparse_veg_bedrock
sparse_veg_till
tussock_gram
wet_sedge
wetlands
AIC
177.5
175.0
172.5
radius
0
2500
5000
7500
10000

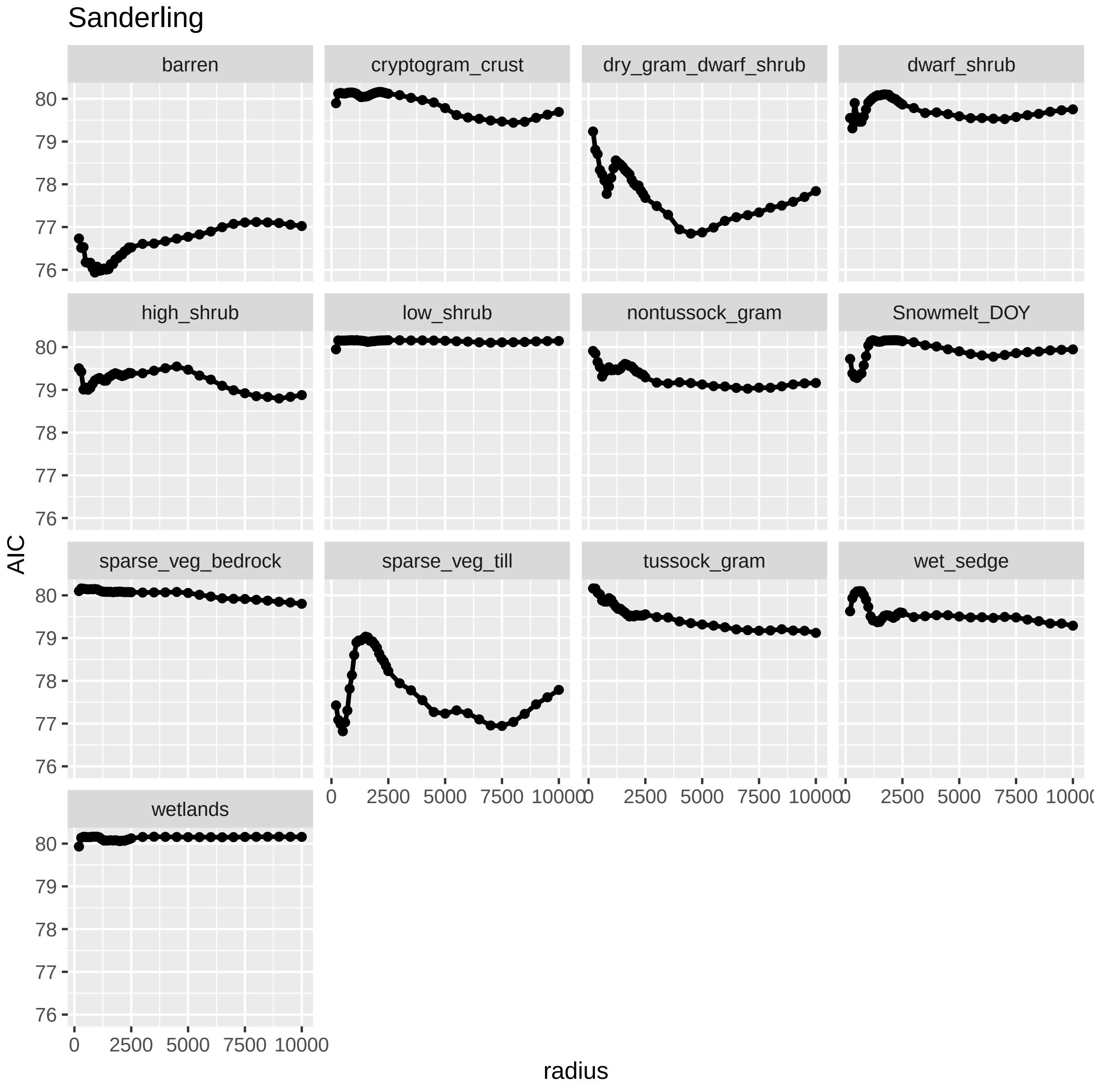

Sanderling
barren
cryptogram_crust
dry_gram_dwarf_shrub
dwarf_shrub
high_shrub
low_shrub
nontussock_gram
Snowmelt_DOY
sparse_veg_bedrock
sparse_veg_till
tussock_gram
wet_sedge
wetlands
AIC
76
77
78
79
80
radius
0
2500
5000
7500
10000

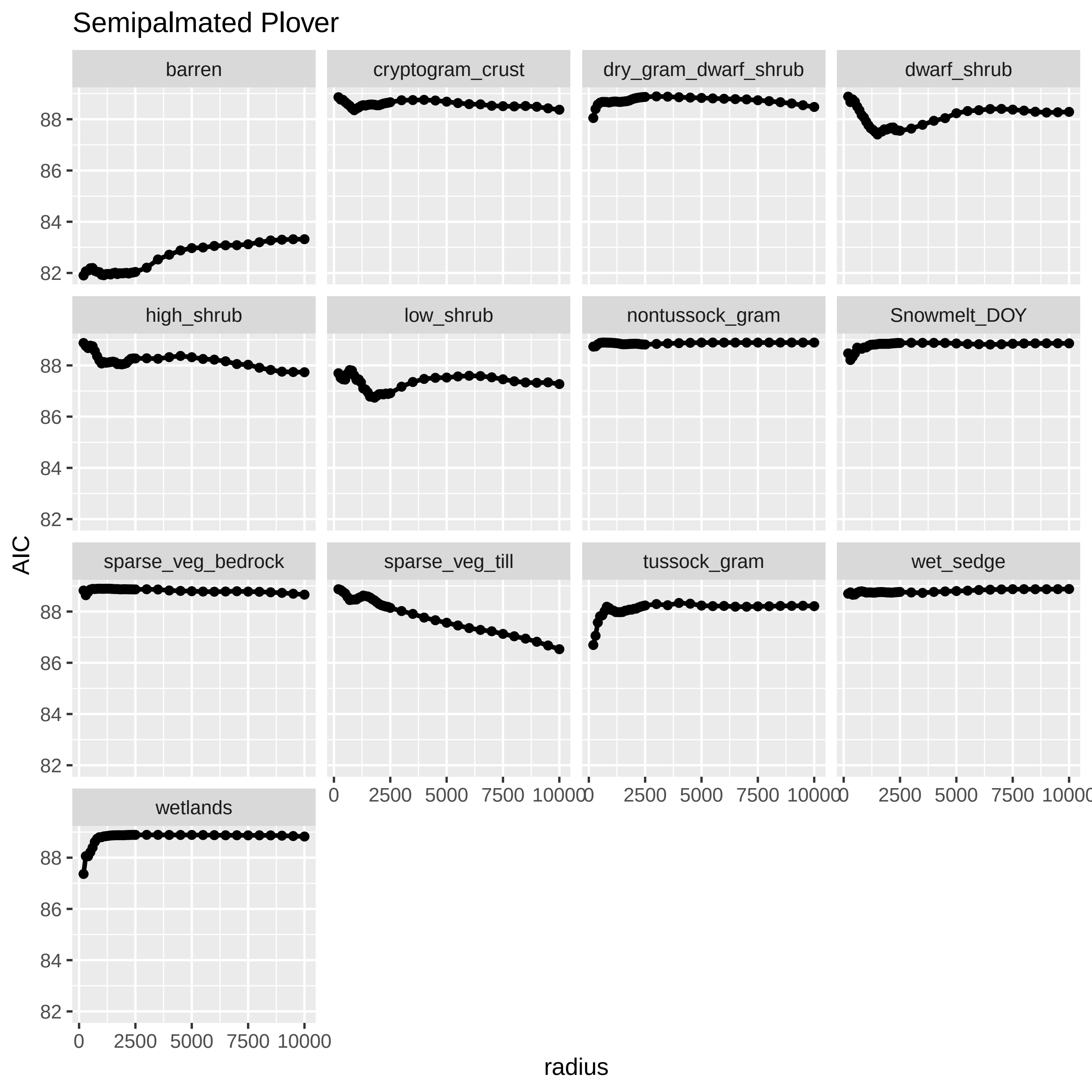

Semipalmated Plover
barren
cryptogram_crust
dry_gram_dwarf_shrub
dwarf_shrub
high_shrub
low_shrub
nontussock_gram
Snowmelt_DOY
sparse_veg_bedrock
sparse_veg_till
tussock_gram
wet_sedge
wetlands
AIC
82
84
86
88
radius
0
2500
5000
7500
10000

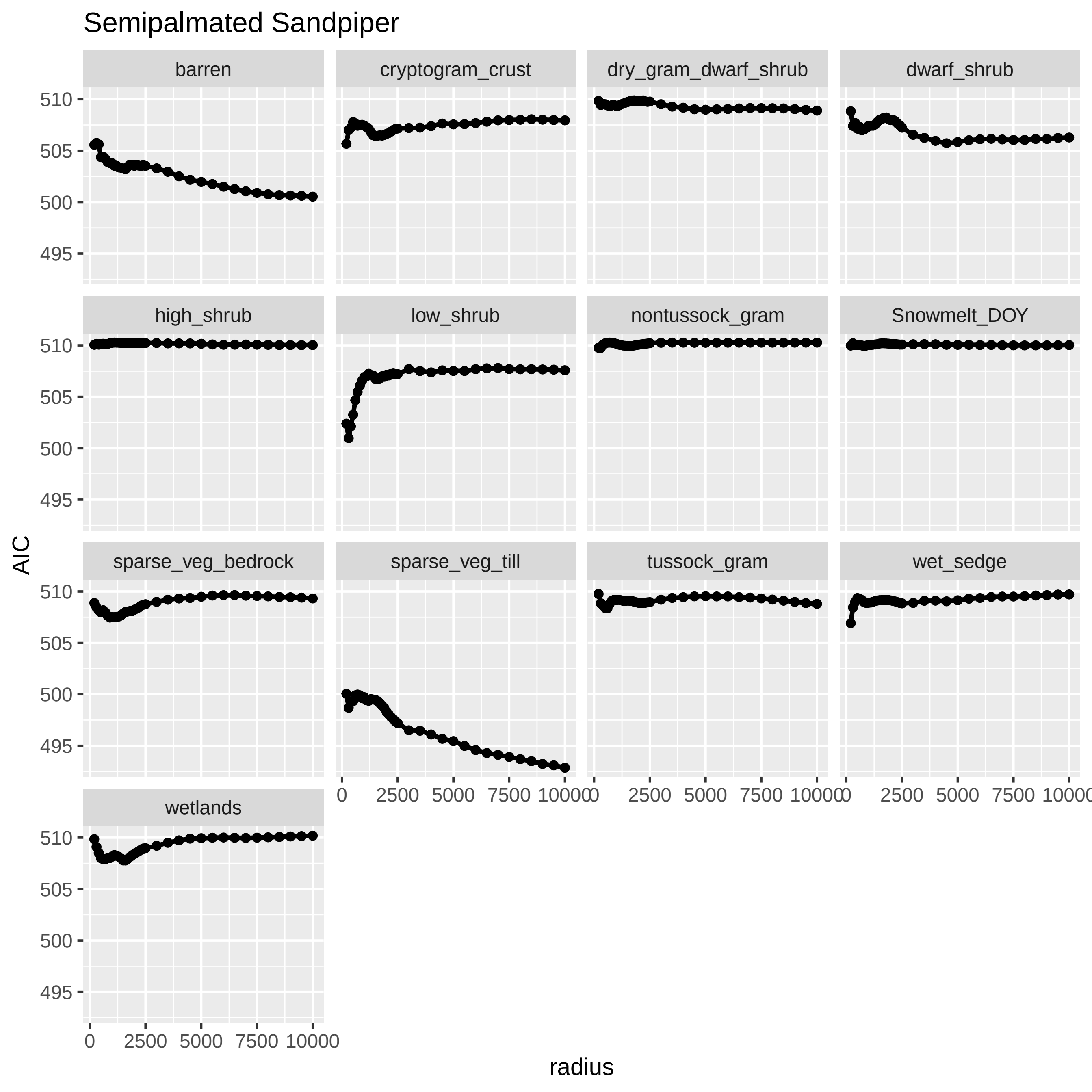

Semipalmated Sandpiper
barren
cryptogram_crust
dry_gram_dwarf_shrub
dwarf_shrub
high_shrub
low_shrub
nontussock_gram
Snowmelt_DOY
sparse_veg_bedrock
sparse_veg_till
tussock_gram
wet_sedge
wetlands
AIC
510
505
500
495
radius
0
2500
5000
7500
10000

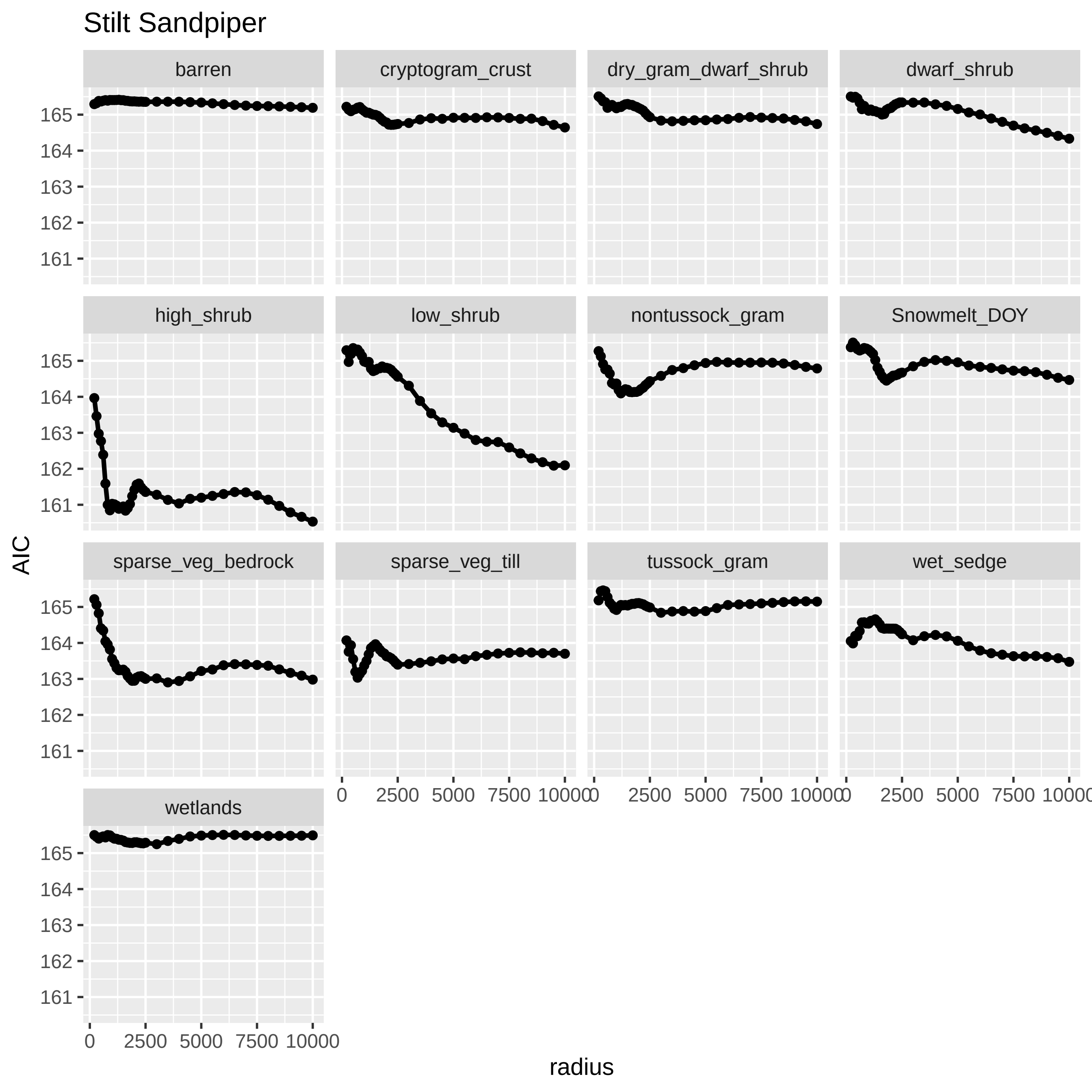
Stilt Sandpiper
barren
cryptogram_crust
dry_gram_dwarf_shrub
dwarf_shrub
high_shrub
low_shrub
nontussock_gram
Snowmelt_DOY
sparse_veg_bedrock
sparse_veg_till
tussock_gram
wet_sedge
wetlands
AIC
161
162
163
164
165
radius
0
2500
5000
7500
10000

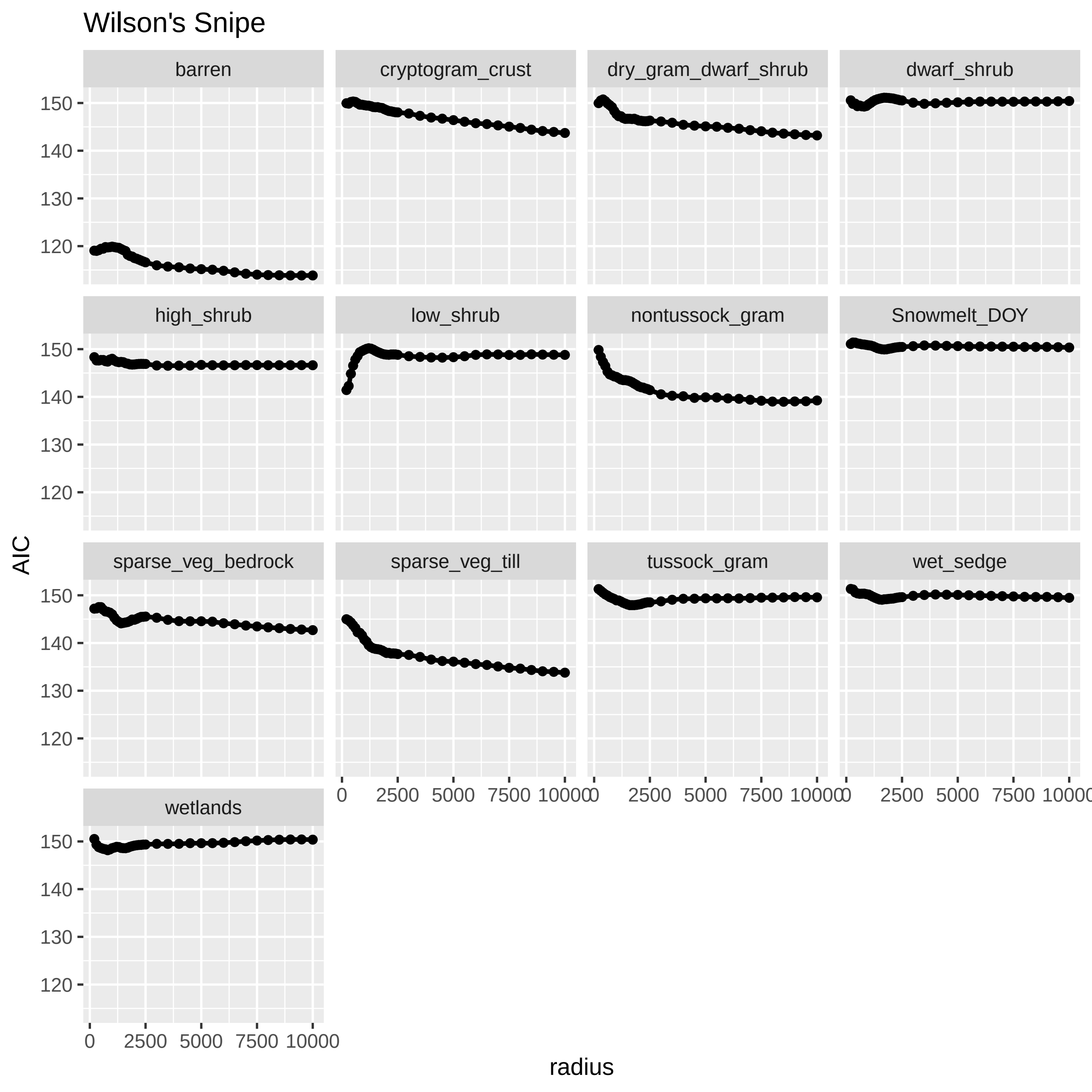

Wilson's Snipe
barren
cryptogram_crust
dry_gram_dwarf_shrub
dwarf_shrub
high_shrub
low_shrub
nontussock_gram
Snowmelt_DOY
sparse_veg_bedrock
sparse_veg_till
tussock_gram
wet_sedge
wetlands
AIC
120
130
140
150
radius
0
2500
5000
7500
10000

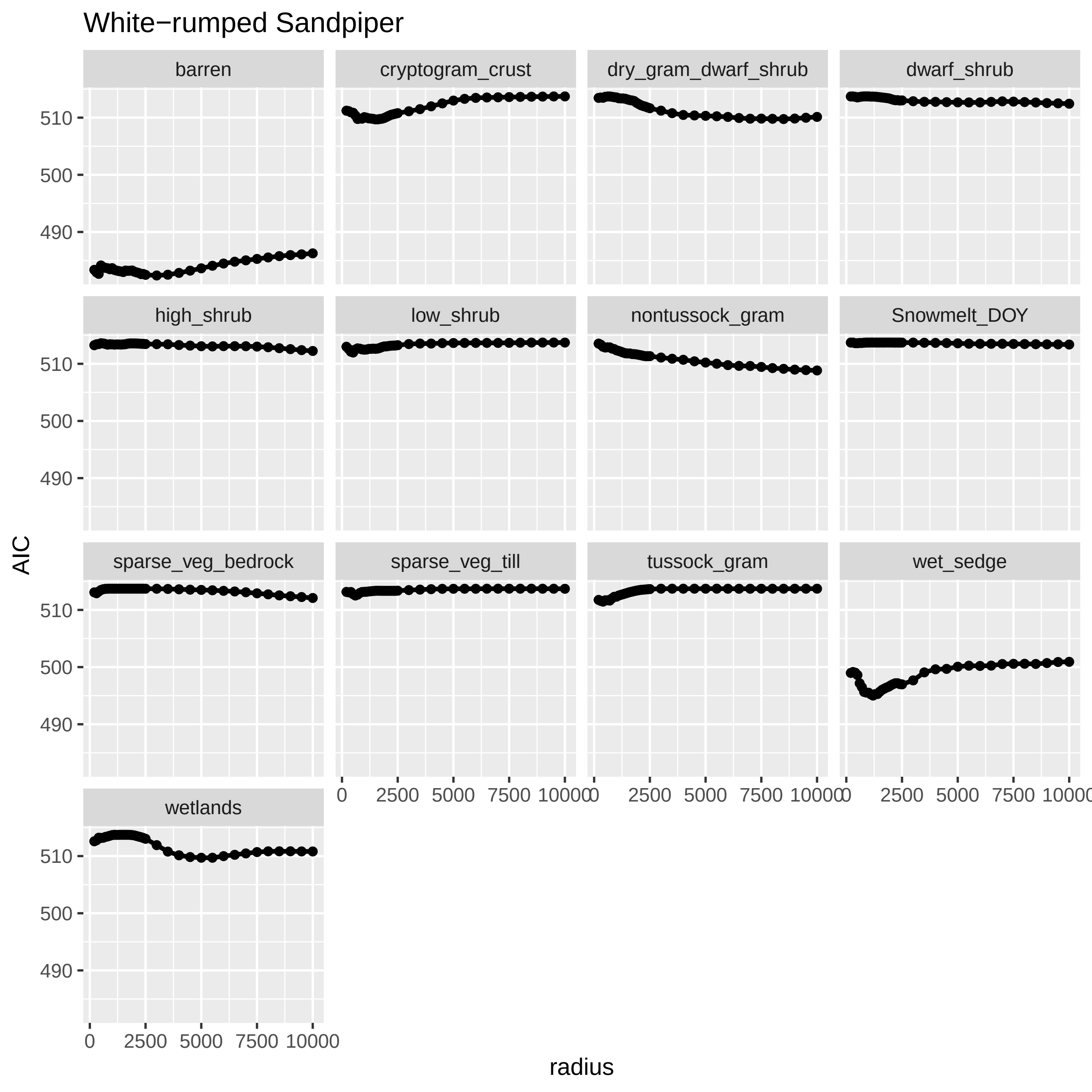
White−rumped Sandpiper
barren
cryptogram_crust
dry_gram_dwarf_shrub
dwarf_shrub
high_shrub
low_shrub
nontussock_gram
Snowmelt_DOY
sparse_veg_bedrock
sparse_veg_till
tussock_gram
wet_sedge
wetlands
AIC
490
500
510
radius
0
2500
5000
7500
10000
