# Supplementary material for: Climate variables are not the dominant predictor of Arctic shorebird distributions
Source: PLoS One. 2023 May 17;18(5):e0285115. doi: 10.1371/journal.pone.0285115 (PMC10191349; doi:10.1371/journal.pone.0285115)
Supplement: S1 Table — See [29] for map of survey regions. (DOCX) [file pone.0285115.s003.docx]

S1 Table. Years in which each PRISM region was surveyed. See (29) for map of survey regions.

|  | Region |  |  |  |  |  |  |  |  |  |  |  |
| --- | --- | --- | --- | --- | --- | --- | --- | --- | --- | --- | --- | --- |
| Year | 1 | 2 | 3 | 4 | 5 | 6 | 7 | 8 | 9 | 10 | 11 | 12 |
| 1994 |  |  |  |  |  |  | X |  | X |  |  |  |
| 1995 |  |  |  |  |  |  | X |  | X |  |  |  |
| 1996 |  |  | X |  |  |  |  |  |  |  |  |  |
| 1997 |  |  | X |  |  |  |  |  |  |  |  |  |
| 2001 |  |  |  |  |  |  |  | X | X | X |  |  |
| 2002 | X |  |  |  |  |  |  | X |  |  |  |  |
| 2003 |  |  | X | X |  | X |  |  |  |  |  |  |
| 2004 |  |  | X | X |  | X |  |  |  |  |  |  |
| 2005 |  |  |  |  |  | X |  | X |  |  |  | X |
| 2006 |  |  |  | X |  | X | X | X |  |  |  | X |
| 2007 |  |  |  |  |  | X | X | X |  | X |  | X |
| 2008 |  |  |  |  |  | X |  |  |  |  |  | X |
| 2009 |  |  |  |  |  | X |  |  |  |  |  | X |
| 2010 |  |  |  |  |  | X | X | X |  |  |  |  |
| 2011 |  |  |  |  |  | X | X | X | X | X |  |  |
| 2012 |  | X |  |  |  | X | X | X | X |  |  |  |
| 2013 |  | X |  |  |  |  |  | X |  | X | X |  |
| 2014 |  |  |  |  |  |  |  | X |  | X |  |  |
| 2015 |  |  |  |  |  | X | x | X |  |  |  |  |
| 2016 | X |  |  |  | X | X |  |  |  |  |  |  |
| 2017 |  | X |  |  |  | X | x |  |  |  |  |  |
| 2018 |  | X |  |  |  |  |  |  |  |  |  |  |
| N yrs | 2 | 4 | 4 | 3 | 1 | 13 | 9 | 11 | 5 | 5 | 1 | 5 |
