# Supplementary material for: Climate variables are not the dominant predictor of Arctic shorebird distributions
Source: PLoS One. 2023 May 17;18(5):e0285115. doi: 10.1371/journal.pone.0285115 (PMC10191349; doi:10.1371/journal.pone.0285115)
Supplement: S2 Table — (DOCX) [file pone.0285115.s004.docx]

| **Predictor** | **Mean** | **SD** | **Min** | **Max** | **Range** |
| --- | --- | --- | --- | --- | --- |
| % Barren | 0.04 | 0.09 | 0 | 1 | 1 |
| % Cryptogamic Crust | 0.04 | 0.05 | 0 | 0.84 | 0.84 |
| % Dry Graminoid Dwarf Shrub | 0.06 | 0.04 | 0 | 0.41 | 0.41 |
| % Dwarf Shrub | 0.09 | 0.1 | 0 | 0.97 | 0.97 |
| % High Shrub | 0.06 | 0.06 | 0 | 0.99 | 0.99 |
| % Low Shrub | 0.09 | 0.12 | 0 | 1 | 1 |
| % Non-tussock Graminoid | 0.08 | 0.07 | 0 | 0.96 | 0.96 |
| % Sparsely Vegetated Bedrock | 0.06 | 0.07 | 0 | 1 | 1 |
| % Sparsely Vegetated Till | 0.08 | 0.11 | 0 | 1 | 1 |
| % Tussock Graminoid | 0.07 | 0.1 | 0 | 0.97 | 0.97 |
| % Wet Sedge | 0.11 | 0.13 | 0 | 0.99 | 0.99 |
| % Wetlands | 0.06 | 0.14 | 0 | 1 | 1 |
| Date of Snowmelt (Day of Year) | 168 | 12 | 143 | 207 | 64 |
| Distance to Coast (km) | 39 | 60 | 1 | 360 | 359 |
| Elevation (m) | 138 | 128 | 3 | 904 | 901 |
| SD elevation (m) | 20 | 22 | 0 | 169 | 169 |
| Mean Annual Temperature (°C) | -12.7 | 3.2 | -21.1 | -1.4 | 19.7 |
| Temperature Seasonality SD (°C) | 15.1 | 1.2 | 1.2 | 17.7 | 16.5 |
| Mean Diurnal Range (°C) | 6.9 | 1 | 1.3 | 9.3 | 8 |
| Annual Precipitation (mm) | 200 | 77 | 64 | 461 | 397 |
| Precipitation Seasonality CV (%) | 63 | 8 | 36 | 89 | 53 |

S2 Table. Range of values for environmental predictors.
