# Supplementary material for: Climate variables are not the dominant predictor of Arctic shorebird distributions
Source: PLoS One. 2023 May 17;18(5):e0285115. doi: 10.1371/journal.pone.0285115 (PMC10191349; doi:10.1371/journal.pone.0285115)
Supplement: S3 Table — (DOCX) [file pone.0285115.s005.docx]

S3 Table. Radius (m) of the optimal scale of effect at which land cover affects shorebird occupancy, determined by AIC

| **Species** | **Barren** | **Cryptogram Crust** | **Dry Graminoid Dwarf Shrub** | **Dwarf Shrub** | **High Shrub** | **Low Shrub** | **Non-tussock Graminoid** | **Sparsely Vegetated Bedrock** | **Sparsely Vegetated Till** | **Tussock Graminoid** | **Wet Sedge** | **Wetlands** | **Snowmelt DOY** |
| --- | --- | --- | --- | --- | --- | --- | --- | --- | --- | --- | --- | --- | --- |
| American  Golden-plover | 200 | 4000 | 500 | 7000 | 1000 | 900 | 6000 | 5000 | 10000 | 200 | 10000 | 300 | 200 |
| Baird’s Sandpiper | 200 | 10000 | 10000 | 2000 | 1800 | 10000 | 2400 | 2200 | 200 | 5500 | 200 | 200 | 10000 |
| Black-bellied  Plover | 7500 | 9500 | 10000 | 9500 | 10000 | 3000 | 10000 | 10000 | 5000 | 1000 | 200 | 3500 | 400 |
| Buff-breasted  Sandpiper | 6000 | 9500 | 2500 | 9500 | 3000 | 2500 | 1400 | 10000 | 800 | 500 | 10000 | 1300 | 200 |
| Dunlin | 2400 | 10000 | 3500 | 5500 | 800 | 200 | 6500 | 300 | 1200 | 200 | 3000 | 3500 | 200 |
| Least Sandpiper | 3500 | 6000 | 10000 | 200 | 5500 | 10000 | 10000 | 700 | 7000 | 2500 | 300 | 3500 | 5500 |
| Pectoral  Sandpiper | 500 | 9000 | 10000 | 2500 | 200 | 1100 | 10000 | 5500 | 3000 | 3000 | 300 | 700 | 200 |
| Red Knot | 1000 | 10000 | 2400 | 2500 | 2400 | 2400 | 2400 | 2500 | 10000 | 3000 | 10000 | 500 | 10000 |
| Red Phalarope | 600 | 200 | 10000 | 3500 | 500 | 10000 | 4500 | 10000 | 600 | 1100 | 700 | 3500 | 200 |
| Red-necked  Phalarope | 10000 | 10000 | 700 | 4000 | 2000 | 4500 | 200 | 6000 | 10000 | 6000 | 200 | 600 | 300 |
| Ruddy Turnstone | 5000 | 400 | 10000 | 200 | 300 | 10000 | 10000 | 10000 | 8000 | 4000 | 500 | 6000 | 500 |
| Sanderling | 8000 | 4500 | 300 | 9000 | 200 | 7000 | 500 | 10000 | 500 | 10000 | 10000 | 200 | 900 |
| Semipalmated  Plover | 900 | 200 | 1500 | 10000 | 1800 | 200 | 300 | 300 | 10000 | 200 | 400 | 200 | 200 |
| Semipalmated Sandpiper | 200 | 10000 | 4500 | 9500 | 300 | 300 | 800 | 900 | 10000 | 600 | 200 | 1600 | 200 |
| Stilt Sandpiper | 10000 | 10000 | 10000 | 10000 | 9500 | 1200 | 1800 | 3500 | 700 | 3000 | 10000 | 3000 | 200 |
| Wilson’s Snipe | 10000 | 10000 | 800 | 3500 | 200 | 8500 | 1700 | 10000 | 10000 | 1600 | 1600 | 800 | 9500 |
| White-rumped  Sandpiper | 1500 | 8500 | 10000 | 10000 | 500 | 10000 | 10000 | 10000 | 600 | 400 | 1200 | 5500 | 200 |
