# Supplementary material for: Climate variables are not the dominant predictor of Arctic shorebird distributions
Source: PLoS One. 2023 May 17;18(5):e0285115. doi: 10.1371/journal.pone.0285115 (PMC10191349; doi:10.1371/journal.pone.0285115)
Supplement: Attachment — Submitted filename: PONE-D-23-03560_marked.pdf [file pone.0285115.s009.pdf]

PLOS ONE

Climate variables are not the dominant predictor of Arctic shorebird distributions

--Manuscript Draft--

**Manuscript Number:**	PONE-D-23-03560
**Article Type:**	Research Article
**Full Title:**	Climate variables are not the dominant predictor of Arctic shorebird distributions
**Short Title:**	Climate variables are not the dominant predictor of Arctic shorebird distributions
**Corresponding Author:**	Christine M. Anderson<br>Carleton University<br>Ottawa, ON CANADA
**Keywords:**	shorebird; wader; arctic; polar; migratory birds; habitat suitability model; species distribution model; environmental niche model; habitat; climate; species range
**Abstract:**	Competing theoretical perspectives about whether or not climate is the dominant factor influencing species' distributions at large spatial scales have important consequences when habitat suitability models are used to address conservation problems. In this study, we tested how much variables in addition to climate help to explain habitat suitability for Arctic-breeding shorebirds. To do this we model species occupancy using path analyses, which allow us to estimate the indirect effects of climate on other predictor variables, such as land cover. We also use deviance partitioning to quantify the total relative importance of climate versus additional predictors in explaining species occupancy. We found that individual land cover variables are often stronger predictors than the direct and indirect effects of climate combined. In models with both climate and additional variables, on average the additional variables accounted for 57% of the explained deviance, independent of shared effects with the climate variables. Our results support the idea that climate-only models may offer incomplete descriptions of current and future habitat suitability, and can lead to incorrect conclusions with important management implications.
**Order of Authors:**	Christine M. Anderson
	Lenore Fahrig
	Jennie Rausch
	Paul A. Smith
**Additional Information:**	
**Question**	**Response**
**Financial Disclosure**<br><br>Enter a financial disclosure statement that describes the sources of funding for the work included in this submission. Review the submission guidelines for detailed requirements. View published research articles from *PLOS ONE* for specific examples.<br><br>This statement is required for submission and **will appear in the published article** if the submission is accepted. Please make sure it is accurate.	The funders had no role in study design, data collection and analysis, decision to publish, or preparation of the manuscript.<br><br>PAS - P57 - ArcticNet - https://arcticnet.ulaval.ca<br>PAS - 2017-06391 - National Science and Engineering Research Council of Canada - https://www.nserc-crsng.gc.ca/index_eng.asp<br><br>PAS and JR - Institutional funding - Environment and Climate Change Canada - https://www.canada.ca/en/environment-climate-change.html<br><br>CA - 518225-2018 - National Science and Engineering Research Council of Canada - https://www.nserc-crsng.gc.ca/index_eng.asp<br><br>CA - W. Garfield Weston Award in Northern Research (Doctoral) 2019 – 2020 - The W. Garfield Weston Foundation. - https://westonfoundation.ca/northern-science-and-knowledge/

Powered by Editorial Manager® and ProduXion Manager® from Aries Systems Corporation

**Unfunded studies**<br>Enter: *The author(s) received no specific funding for this work.*<br><br>**Funded studies**<br>Enter a statement with the following details:<br>• Initials of the authors who received each award<br>• Grant numbers awarded to each author<br>• The full name of each funder<br>• URL of each funder website<br>• Did the sponsors or funders play any role in the study design, data collection and analysis, decision to publish, or preparation of the manuscript?<br>• **NO** - Include this sentence at the end of your statement: *The funders had no role in study design, data collection and analysis, decision to publish, or preparation of the manuscript.*<br>• **YES** - Specify the role(s) played.<br><br>* typeset	
**Competing Interests**<br><br>Use the instructions below to enter a competing interest statement for this submission. On behalf of all authors, disclose any competing interests that could be perceived to bias this work—acknowledging all financial support and any other relevant financial or non-financial competing interests.<br><br>This statement is required for submission and **will appear in the published article** if the submission is accepted. Please make sure it is accurate and that any funding sources listed in your Funding Information later in the submission form are also declared in your Financial Disclosure statement.<br><br>View published research articles from *PLOS ONE* for specific examples.	The authors have declared that no competing interests exist.

**NO authors have competing interests**<br><br>Enter: *The authors have declared that no competing interests exist.*<br><br>**Authors with competing interests**<br><br>Enter competing interest details beginning with this statement:<br><br>*I have read the journal's policy and the authors of this manuscript have the following competing interests: [insert competing interests here]*<br><br>* typeset	
**Ethics Statement**<br><br>Enter an ethics statement for this submission. This statement is required if the study involved:<br><br>• Human participants<br>• Human specimens or tissue<br>• Vertebrate animals or cephalopods<br>• Vertebrate embryos or tissues<br>• Field research<br><br>Write "N/A" if the submission does not require an ethics statement.<br><br>General guidance is provided below. Consult the submission guidelines for detailed instructions. **Make sure that all information entered here is included in the Methods section of the manuscript.**	Surveys were observational, and involve no capture or sampling. Lands surveyed include Federal Crown Lands, Inuit Owned Lands, and Federal and Territorial Protected Areas. Access to these lands was granted through permits to JR including: Environment and Climate Change Canada (NWT-MBS-17-03, MM-NR-2022-NU-005, NUN-NWA-17-04, SC-NR-2001-NT-004, SC-NR-2022-NU-005), Government of Nunavut (WL2022-031), Kitikmeot Inuit Association (KTX115X007), Kivalliq Inuit Association (KVX15N05), Qikiqtani Inuit Association (Q14X001).

Powered by Editorial Manager® and ProduXion Manager® from Aries Systems Corporation

**Format for specific study types**<br><br>**Human Subject Research (involving human participants and/or tissue)**<br>• Give the name of the institutional review board or ethics committee that approved the study<br>• Include the approval number and/or a statement indicating approval of this research<br>• Indicate the form of consent obtained (written/oral) or the reason that consent was not obtained (e.g. the data were analyzed anonymously)<br><br>**Animal Research (involving vertebrate animals, embryos or tissues)**<br>• Provide the name of the Institutional Animal Care and Use Committee (IACUC) or other relevant ethics board that reviewed the study protocol, and indicate whether they approved this research or granted a formal waiver of ethical approval<br>• Include an approval number if one was obtained<br>• If the study involved *non-human primates*, add *additional details* about animal welfare and steps taken to ameliorate suffering<br>• If anesthesia, euthanasia, or any kind of animal sacrifice is part of the study, include briefly which substances and/or methods were applied<br><br>**Field Research**<br><br>Include the following details if this study involves the collection of plant, animal, or other materials from a natural setting:<br>• Field permit number<br>• Name of the institution or relevant body that granted permission	
**Data Availability**<br><br>Authors are required to make all data underlying the findings described fully available, without restriction, and from the time of publication. PLOS allows rare exceptions to address legal and ethical concerns. See the PLOS Data Policy and FAQ for detailed information.	Yes - all data are fully available without restriction

A Data Availability Statement describing where the data can be found is required at submission. Your answers to this question constitute the Data Availability Statement and **will be published in the article**, if accepted.<br><br>**Important:** Stating 'data available on request from the author' is not sufficient. If your data are only available upon request, select 'No' for the first question and explain your exceptional situation in the text box.<br><br>Do the authors confirm that all data underlying the findings described in their manuscript are fully available without restriction?	
**Describe where the data may be found in full sentences. If you are copying our sample text, replace any instances of XXX with the appropriate details.**<br><br>• If the data are **held or will be held in a public repository**, include URLs, accession numbers or DOIs. If this information will only be available after acceptance, indicate this by ticking the box below. For example: *All XXX files are available from the XXX database (accession number(s) XXX, XXX.)*.<br>• If the data are all contained **within the manuscript and/or Supporting Information files**, enter the following: *All relevant data are within the manuscript and its Supporting Information files.*<br>• If neither of these applies but you are able to provide **details of access elsewhere**, with or without limitations, please do so. For example:<br><br>*Data cannot be shared publicly because of [XXX]. Data are available from the XXX Institutional Data Access / Ethics Committee (contact via XXX) for researchers who meet the criteria for access to confidential data.*<br><br>*The data underlying the results presented in the study are available from (include the name of the third party*	All data and code files are available from the Zenodo database (https://doi.org/10.5281/zenodo.7613304).

and contact information or URL).<br>• This text is appropriate if the data are owned by a third party and authors do not have permission to share the data.<br><br>* typeset	
Additional data availability information:	

Powered by Editorial Manager® and ProduXion Manager® from Aries Systems Corporation

Manuscript

Click here to access/download;Manuscript;Main file_Shorebird habitat suitabililty.docx

Climate variables are not the dominant predictor of Arctic shorebird distributions

Christine M. Anderson [1*], Lenore Fahrig [1], Jennie Rausch [2], Paul A. Smith [3]

[1] Department of Biology, Geomatics and Landscape Ecology Laboratory, Carleton University, Ottawa, ON, Canada

[2] Canadian Wildlife Service, Environment and Climate Change Canada, Yellowknife, NT, Canada

[3] Wildlife Research Division, Environment and Climate Change Canada, Ottawa, ON, Canada

Abstract

Competing theoretical perspectives about whether or not climate is the dominant factor influencing species' distributions at large spatial scales have important consequences when habitat suitability models are used to address conservation problems. In this study, we tested how much variables in addition to climate help to explain habitat suitability for Arctic-breeding shorebirds. To do this we model species occupancy using path analyses, which allow us to estimate the indirect effects of climate on other predictor variables, such as land cover. We also use deviance partitioning to quantify the total relative importance of climate versus additional predictors in explaining species occupancy. We found that individual land cover variables are often stronger predictors than the direct and indirect effects of climate combined. In models with both climate and additional variables, on average the additional variables accounted for 57% of the explained deviance, independent of shared effects with the climate variables. Our results support the idea that climate-only models may offer incomplete descriptions of current and future habitat suitability, and can lead to incorrect conclusions with important management implications.

Introduction

Habitat suitability models are used to describe the relationship between species' presence in geographic space and the characteristics of the environment, and to estimate how likely a species is to occupy unsampled locations. Along with closely related species distribution models, environmental niche models, resource selection functions and bioclimatic envelope models [but see 1], these analyses are increasingly popular for addressing a wide range of questions, such as how species will respond to climate change [e.g. 2], identifying priority areas for threatened species [e.g. 3], managing landscapes [e.g. 4], and understanding the spread of invasive species [e.g. 5]. However, different theoretical perspectives about how these relationships between species and their environment are structured have important consequences for the variables included in these models, and therefore how we use them to address conservation problems.

One hypothesis suggests that climate is the dominant factor influencing species' distributions at large spatial scales [6,7]. Climate influences species' distributions directly, for example through thermal tolerances, and indirectly, for example through climate's influence on land cover (Fig 1). Interestingly, many of the papers that invoke this hypothesis are studies of future species distribution based on climate-only models [i.e. 8]. One reason for this practice is pragmatic: often there are no future predictions available for the other potential covariates of distribution such as land cover [9]. However, these papers also often argue that at large spatial extents, such as entire ranges for widespread species, the direct and indirect effects of climate encompass the majority of the influences on species' distributions [10]. 'Climate-only' models are often evaluated using a threshold, such as a model with an AUC of at least 0.7 has "good" explanatory power, and usually climate-only models exceed the threshold [i.e. 11,12].

Fig 1. Illustration of the common assumption that, over large spatial extents, climate predictors drive the probability of species occupancy. Such climate-only models imply that the total effects of climate predictors, i.e., both their direct effects (a) and their indirect effects through their effects on additional predictors such as land cover (b*c), are much greater than the direct effects of these additional predictors (c). The underlying assumption is that climate is the main driver of variation in the predictors that influence species occupancy, and so it is only necessary to include climate predictors in species occupancy models.

A competing hypothesis suggests that habitat suitability models need to be more comprehensive because there can be additional important factors shaping species distributions that may not be well described as an indirect effect of climate, as is often assumed in climate-only models. In contrast to the future-oriented models described above, papers estimating current species distributions often include a wide range of predictors, many of which are non-climatic, relating instead to resources and habitat structure [13]. Studies aimed at estimating current habitat suitability typically compare multiple models to identify a best model, which is rarely a climate-only model [i.e. 14–16]. In particular, biotic interactions may be important even at large spatial extents, not just at local scales [17]. For example, many species require the presence of specific plants as food or hosts to facilitate their presence in a region [18]. Precipitation is often a poor proxy for the water available to plants, as topography and soil substrate control how precipitation translates into soil moisture [19]. Soil predictors such as pH and nutrients are important predictors of plant distribution, are strongly influenced by underlying geology, irrespective of climate [20].

Here, we ask how much do variables in addition to climate help to explain habitat suitability? To do this we model species occupancy using path analyses, which allow us to estimate the indirect effects of climate on other predictor variables, such as land cover. We also use partitioning to quantify the total relative importance of climate versus additional predictors in explaining species occupancy. We addressed our question using data on the distribution of Arctic-breeding shorebirds. Most papers studying habitat suitability for Arctic-breeding shorebirds have been conducted at smaller extents, and for the most part they do not include climate variables, likely because climate does not vary enough over smaller extents to be a relevant predictor [i.e., 21–23]. The few large-extent models that have been used to predict habitat suitability for these species include a model that largely relies on climate predictors [24], but also a report suggesting that additional predictors beyond climate were important for predicting current and future occupancy of Red Knot (*Calidris canutus)* and Semipalmated Sandpipers (Calidris pusilla; 25). Based on previous studies of Arctic shorebird habitat associations, the additional predictors we consider are land cover, snow cover, substrate chemistry, and elevation, and the standard deviation of elevation, as shorebirds prefer flat habitats [21,24,26,27].

A priori, we expected that the probability of shorebird occupancy at sites distributed over large spatial extents can be estimated considerably better by explicitly including additional variables in habitat suitability models, rather than assuming that these additional variables are themselves driven by climate. If climate is the dominant factor influencing the occupancy of breeding shorebirds across the Arctic, we expect that the variation in shorebird occupancy is mostly explained by the combination of the direct effects of predictors from climate-only models and their indirect effects through their effects on the additional predictors. If, independent of climate, additional predictors have important influences on the occupancy of breeding shorebirds across the Arctic, we expect that a substantial amount of the explained variation in shorebird occupancy is uniquely attributed to the additional predictors not found in climate-only models.

Methods

Overview

To look at the predictors of shorebird occupancy, their interrelationships, and their relative effect sizes, we developed a hypothetical path diagram outlining our hypotheses about how climate predictors and additional environmental predictors are interrelated (Fig 2). We identified predictors that have been important in other models of habitat suitability for Arctic-breeding shorebirds [21,24,26,27]. We used path analysis to verify the structure of our path diagram and assess the degree to which the effects of additional predictors are driven by climate. We then used a deviance partitioning approach to test whether the total (direct + indirect) effects of climate predictors explained most of the variation in shorebird occupancy. All analyses were performed using R Statistical Software (v4.2.1; 28).

Fig 2. Path diagram outlining the hypothesized relationships among predictors of shorebird occupancy. Groups of predictors with a blue label are included in climate-only models, and groups of predictors with an orange label are additional predictors included in climate+additional models. Arrows indicate direct causal paths between all combinations of variables in the respective groups are included in the path model of occupancy for each shorebird species. For example, the arrow from climate to land cover represents 60 arrows, one from each climate variable to each land cover variable.

Shorebird Surveys

Plots were surveyed for shorebirds across the Canadian Arctic as part of the Arctic Program for Regional and International Shorebird Monitoring (PRISM; 29), which covers all of Arctic North America as defined by the Circumpolar Arctic Vegetation Map [30]. The Canadian PRISM data used here includes 2336 plots across the Canadian Arctic (Fig 3), an area of 3.5 million km^2. Given this massive area, surveys were completed over a number of years, from 1994-2018, with a majority of the sampling effort concentrated between 2003 and 2018. The Canadian Arctic was divided into 12 regions based on logistical considerations. Within each region plots were selected by random sampling, stratified by habitat. The proportion of plots in wet, moist and dry habitats varied by region depending on habitat abundance [see 29 for more details], but on average 16%, 29%, and 56% of plots were in wet, moist, and dry habitats respectively. In all years, surveys were conducted between June 18 and July 15. This corresponds with the latter part of shorebird courtship and the early part of incubation, when breeding territories could be identified from the birds' territorial displays. Plots were typically 12-16 ha (400 m x 300-400 m). We excluded one set of surveys because extensive flooding led to very atypical breeding conditions. We also excluded surveys for which observers recorded nearby human development, which is rare across the study area and could potentially alter shorebird habitat preferences. For 211 plots that were visited in more than one year, we randomly selected one of the surveys to be included in the final dataset.

Excluding this is likely fine for this analysis, but could you could also have included to first see if it was an outlier. Might have been informative?

Fig 3. Map of northern Canada showing the locations of plots where breeding shorebirds were surveyed, 1994-2018.

If highest breeding evidence is relevant in this analysis, then add details of the codes/categories or provide a citation. However, if not, then delete this text.

Plots were surveyed for breeding birds following PRISM protocols [29]. Two surveyors searched the plot walking straight-line transects, covering a breadth of 50 m with the observers situated 25 m apart, using a GPS to ensure complete coverage of the entire plot. Surveys took approximately 90 mins. Observers recorded the number and species of all birds observed within each plot, and recorded the highest evidence of breeding. Altogether we observed 19 species of shorebirds, 17 of which were observed in at least 30 plots, our minimum threshold for developing habitat suitability models: American Golden-Plover (*Pluvialus dominica*), Baird's Sandpiper (*Calidris bairdii*), Black-bellied Plover (*Pluvialis squatrola*), Buff-breasted Sandpiper (*Tryngites subruficollis*), Dunlin (*Calidris alpina*), Least Sandpiper (*Calidris minutilla*), Pectoral Sandpiper (*Calidris melanotos*), Red Knot (*Calidris cantus*), Red Phalarope (*Phalaropus fulicarius*), Red-necked Phalarope (*Phalaropus lobatus*), Ruddy Turnstone (*Arenaria interpres*), Sanderling (*Calidris alba*), Semipalmated Sandpiper (*Calidris pusilla)*, Stilt Sandpiper (*Calidris himantopus*), Wilson's Snipe (*Gallinago delicata*), and White-rumped Sandpiper (*Calidris fuscicollis*). Surveys were observational, and involve no capture or sampling. Lands surveyed include Federal Crown Lands, Inuit Owned Lands, and Federal and Territorial Protected Areas. Access to these lands was granted through permits to JR including: Environment and Climate Change Canada (NWT-MBS-17-03, MM-NR-2022-NU-005, NUN-NWA-17-04, SC-NR-2001-NT-004, SC-NR-2022-NU-005), Government of Nunavut (WL2022-031), Kitikmeot Inuit Association (KTX115X007), Kivalliq Inuit Association (KVX15N05), Qikiqtani Inuit Association (Q14X001).

Environmental predictors

We used two categories of environmental predictors of shorebird occupancy: predictors found in 'climate-only models', and additional predictors in 'climate+additional models'. For the climate

predictors we used the bioclimatic data available from WorldClim 2 at a 30 arc-second resolution [31]. This data is typical of that used in climate-only habitat suitability models because it is global in scope and easily accessible. We eliminated any predictors that had correlations higher than 0.7, selecting the 5 uncorrelated climate predictors that seemed most relevant to shorebird occupancy: annual mean temperature, temperature seasonality (standard deviation of mean monthly temperature x 100), mean diurnal range (mean of monthly max temp – min temp), annual precipitation, and precipitation seasonality (coefficient of variation of monthly precipitation).

Additional predictors in the climate+additional models of shorebird occupancy included topography, geology, snowmelt, and land cover variables. The topography predictors we included were elevation, the standard deviation of elevation, and distance to coast. We used elevation data available from WorldClim 2, also at a 30 arc-second resolution. From this, we derived the standard deviation of elevation over a 5km grid. We calculated distance to coast from coastline data available from Natural Earth [32]. To represent geology, we used substrate chemistry data from the Circumpolar Arctic Vegetation map [30], which includes 3 categories: acidic (pH < 5.5), circumneutral (pH 5.5-7.2) and carbonate (pH > 7.2). Snowmelt timing was derived from an 8-day composite MODIS product, and we used the day of 50% snowmelt per 500m pixel [33]. We used land cover data from the Circa-2000 Northern Land Cover Map of Canada, with 15 land cover classes derived from Landsat data at a 30m resolution [34].

You might want to address the variation in resolution b/w your different variables. 30 arc-seconds is circa 1km

Statistical Analysis

Before building models of habitat suitability for each species of shorebird, we first determined the spatial extent at which day of snowmelt and each land cover predictor most strongly affected each species, their "scale of effect" [35]. Habitat selection is scale dependent, therefore identifying the

appropriate scale at which species respond is important for making correct inferences about the relative importance of environmental variables [36]. We did not complete this step for climate, topography or substrate because these variables were measured at much larger spatial resolutions, and show little variation at small spatial resolutions. To find the scales of effect, we first filtered the plots to a reduced set that were at least 10km from each other. We calculated the mean day of snowmelt and the proportion of each land cover type at multiple scales, centered on the middle of each plot. We tested spatial scales from a radius of 200m, approximately covering the plot itself, to 10km, increasing by increments of 100m up to 2500m and by increments of 500m thereafter. We created a series of logistic regression models relating the occupancy of each of the 17 shorebird species to each predictor at each spatial scale. Then for each predictor, we identified the scale of effect for that species as the extent where the model had the lowest AIC value. In the models described below, we entered the snowmelt timing and land cover predictors at their scales of effect.

We used path analysis to test whether the effects of the additional variables on habitat suitability for shorebirds were largely explainable as indirect effects of climate. Path models were fit using the R package piecewiseSEM [37]. We fit a path model for each of the 17 shorebird species, following the causal structure illustrated in Fig 2. We also included correlated error terms among all climate variables, and among all land cover variables. All models included an offset for plot size. We used a test of directed separation (d-sep) to evaluate the goodness-of-fit for our hypothesized path model, assessing the conditional independence between pairs of variables in the model. Significant d-sep test results indicated associations between substrate and climate, and substrate and shorebird presence, so we included these links as correlated error terms as well. For each species model, standardized effects and confidence intervals were estimated using nonparametric bootstrapping from 10,000 resamples using the R package semEff [38]. The path coefficients we present are semi-partial correlations, which are the correlation between the unique components of predictors (residualised on

What about time of season?

I assume the semi-partial coefficients are within a bootstrap, but are you basing inference on the median (and 95%CI) across bootstraps?

other predictors) and the response variable. Note that this approach controls for multicollinearity between predictors, whereas many path analyses present standardized partial regression coefficients which do not control for multicollinearity, which often creates an upward bias in perceived effect sizes [39]. Model fit was visually assessed using residual plots. To assess the relative strength of individual predictors, we compared the absolute values of their total effects on shorebird species occupancy. The total effect of a predictor is the sum of its direct effects (the path coefficients between the predictor and the shorebird response) and its indirect effects (the product of the path coefficients between the predictor, intermediate predictors, and the shorebird response). Effects were considered to be significant when 95% confidence intervals did not contain 0.

were the residuals calculated from observed - prediction from median coefficier values? Or did you build a distribution acros all 10K bootstraps? Why not additional measure of predictive accuracy on independent withheld data?

To assess the overall relative contribution of climate-only model predictors and additional predictors to shorebird occupancy, we used a deviance partitioning approach. We used Generalized Linear Models (GLMs) with a binomial distribution to determine the deviance explained by climate and additional predictors. Species were analysed separately. Deviance was partitioned, using the ratio of the null deviance and the deviance explained by one of the following factors: climate predictors, additional predictors, deviance shared between climate and additional predictors, and unexplained deviance.

Results

Very low densities (<5 birds / survey across 17 spp). In methods you might wish to mention that this is why you are modeling this occupancy and not as counts

We observed 11,636 shorebirds in surveys of 2336 plots from 1994-2018. At least one shorebird was observed on 54% of plots. By species, occupancy varied from 1% (Red Knot) to 14% (Semipalmated

AIC selection of scale effect would be good to have in the as supplemental material tables

Sandpiper); the average (±SD) species occupancy was 6 ± 4%. Descriptive statistics of the environmental predictor values are in S2 Table. The mean scale of effect for land cover classes was 4400m (median = 3000m). For virtually all land cover classes, the scale of effect varied from the smallest possible scale (200m, just covering the plot) to the largest possible scale (10km) depending on the species. Similarly, each species responded to the different land cover classes at very different spatial scales (Fig S3). The median scale of effect for snowmelt timing was 5000m (median = 3500m)

I don't see an S3 Fig. Only S1 Fig.

Direct effects of climate-only model predictors accounted for on average 17% of the total explained deviance in shorebird occupancy (Fig 4). The total effect of climate-model predictors (direct effect + shared/indirect effect) accounted for on average 43% of the total explained deviance in shorebird occupancy. The unique effects of additional predictors included in climate+additional models, beyond the indirect effects of climate through these predictors, accounted for on average 57% of the total explained deviance in shorebird occupancy (**Error! Reference source not found.**). Depending on the species, the ratio of deviance explained by climate-only predictors vs additional predictors ranged from 80% climate/20% additional to 10% climate/90% additional. The mean explained deviance for our models of shorebird occupancy was 27% (ranging from 9%-42%). The mean AUC of our models of shorebird occupancy was 0.87 (ranging from 0.72-0.93). The full results of the GLMs used for deviance partitioning can be found in S4 Table.

Fig 4. Deviance partitioning for GLMs predicting the occupancy of each of 17 shorebird species. Each bar represents the total deviance explained for each species. The direct effects of climate, the proportion of explained deviance attributed to predictors from the climate-only model, is dark blue. The indirect effects of climate, the proportion of deviance that is explained by both climate and additional variables, is light blue. The total effect of climate on

the occupancy of each species is the sum of the dark and light blue segments. The proportion of explained deviance attributed only to the additional predictors from the climate+additional models is orange. For half of the species shown, the unique effects of the additional variables explain more deviance in shorebird occupancy than the total effect of climate.

Overall, the total effect sizes of climate predictors (direct + indirect sizes; Fig 2) and additional predictors on shorebird occupancy were similar (Fig 5). The 5 predictors with the largest significant absolute effect on shorebird occupancy were additional predictors, specifically the predictors that described the proportion land cover at the species-specific scale of effect of high shrub, dwarf shrub, sparsely vegetated till, dry graminoid dwarf shrub, and non-tussock graminoid. The climate predictors with the largest significant absolute effect on shorebird occupancy were mean diurnal range and mean annual temperature (Fig 5). A summary of the significant total effects of predictors on shorebird occupancy can be found in S5 Table. The full results of the 17 species-specific path models, including summaries of the direct, indirect and total effects of the paths between all variables can be found in S6 Table.

Fig 5. Box and whisker plots showing the median and interquartile range of the absolute total effect sizes of significant (p>0.05) paths from individual path models of occupancy for the 17 most abundant shorebird species in the Canadian Arctic. Survey data was collected from 1994-2018 (Fig. 3). The total effect sizes for each variable are the sum of its direct and indirect effects on shorebird occupancy (Fig. 2). The predictors with the five largest effect sizes were

additional variables. The magnitude of the effect sizes for climate and additional variables fall in a similar range.

I am generally inclined to agree with your approach and conclusions. However, another difference to consider relative to other studies, yours still has fairly restricted sampling and thereby excludes a lot of climate variation that entails structural zeros. I think a lot of studies have strong climate signals because sampling goes FAR beyond the species extant ranges (e.g. for example if you included eBird and BBS with your PRISM data, or if you extent PRISM several hundred KM into the boreal forest) that add climate noise to the data, but are truly structural zeros because they are not even plausible as possible localities of occurrence. Sampling between the 49th parallel and your southernmost location would add a lot of zeros and climate variation. Since a lot of studies mine global datasets, they contain those excess zeros and the climate signal is huge. I am not sure you can fully resolve this here, but should speak to it somewhat as an alternative reason other studies find climate dominates. Perhaps simulation studies perhaps will be needed to resolve how best to approach the issue (should a threshold prob of occurrence be reached before trying to partition climate versus habitat?). Perhaps other papers should consider limiting the distances from known breeding range to better attribute deviance between climate and habitat? Maybe including those wider data to model climate envelopes of species ranges, but restricting spatial extent of contributing data to partition climate vs. habitat is the answer?

Discussion

Our results suggest that the direct and indirect effects of climate variables are not enough to explain the range-wide variation in the occupancy of Arctic-breeding shorebirds. Individual land cover variables are often stronger predictors than the direct and indirect effects of climate combined. In models with climate+additional variables, on average the additional variables accounted for more than half of the explained deviance, independent of shared effects with the climate variables. Our results support the idea that climate-only models may offer incomplete descriptions of current and future habitat suitability, and can lead to incorrect conclusions with important management implications.

Even at broad spatial scales, Arctic-breeding shorebirds typically have distinct land cover preferences that are stronger predictors of habitat suitability than climate, and we show here that land cover is not a simple derivative of climate. Aside from climate, tundra vegetation and soils are also the product of the geological parent material and water drainage [40]. Acidic and non-acidic tundra soils have substantially different biogeochemical fluxes, which in many cases are larger differences than between bioclimatic vegetation zones [40]. These land cover and vegetation characteristics, which are only partly predicted by climate, are well established predictors of shorebird habitat. For example, Dunlin and Red Phalaropes are more likely to be present in in moist, lowland habitats [21], while Red Knots prefer sparsely vegetated habitats [26]. Habitat suitability models for eight shorebird species on the Alaska North Slope showed that all species were much less likely to be breed in upland shrub

habitats [27]. Semipalmated Plovers commonly nest on stony shorelines, which helps them avoid nest predation by Arctic Foxes [51].

is 'to' out of place here?

We should expect to a spectrum of how well habitat suitability for individual species can be predicted by climate. For example, Pöyry et al [41] found that butterflies with high mobility were modelled less accurately by climate-only models than species with low mobility. Species that use a wide range of climates are generally modelled less accurately than species found in a limited range of climatic conditions [42]. The shorebirds studied here breed in the Arctic, and winter in tropical and subtropical areas, encountering vastly different climatic conditions throughout their annual cycle. Here, we saw that within closely related shorebird taxa in the same Arctic ecosystem, the total effect of climate explained anywhere from 10% to 80% of the explained deviance in shorebird occupancy.

In the debate about whether climate-only models are adequate for describing habitat suitability, one important factor that leads different papers to come to different conclusions is the spatial scale at which studies are conducted, which includes both the extent and the grain [43]. Even Pearson and Dawson [10], advocates of climate-only models, recommend that these analyses be undertaken with careful consideration: “...bioclimate envelope models can provide a useful starting point when applied to suitable species and **at appropriate scales**.” However, it seems that whether or not a climate-only model is appropriate for the given species and scale is often assumed rather than tested. For example, Wauchope et al. [24] write “Data were interpolated to a spatial grain size of 10 x 10 km to reflect the approximate resolution of most of the distributional records and a scale at which climate, rather than microhabitat factors, is more likely to be limiting [44]”. Spatial grain has been demonstrated to be important in similar studies. For example, Luoto et al. [45] found that climate-only models of bird distributions in Finland were improved by including land-cover at 10km and 20km resolutions, but not at 40km and 80km resolutions.

Optimizing the scale of effect for land cover and other predictors that can be measured at a range of spatial scales is an important step that is often neglected [36]. Here, we found that Arctic-breeding shorebirds were responding to land cover at a radius of up to 10km, but that the scale of effect differed across species and land cover categories. A single-scale study of vertebrates in Florida [46], contrary to our results, found that including additional predictors had a relatively minor effect on the accuracy of climate-only habitat suitability models. They suggested that the 4km grain of their study may have been too coarse to resolve the land cover associations for their study species, following the hypothesis that biotic interactions are most important at smaller spatial scales. Another important difference between our study and that of Bucklin et al. [46] may be that the spatial extent of our study area was approximately 20x larger; therefore, there may be more available variation in land cover across our sites, increasing its importance. Single-scale studies may therefore miss important predictors, because they are not measuring the predictors at the scale at which species are responding to the predictor [43].

Other reasons that climate-only models may be preferred are purely practical. Although important, additional predictors are often neglected because they are unavailable, or harder to obtain at the extent and resolution needed [20,47]. Particularly for future predictions of habitat suitability, future climate projections are widely available, while other future projections such as land cover are much more limited in their availability, their spatial resolution, their accuracy, and the level of detail that they provide in terms of the number of classification categories [48,49].

We conclude that environmental predictors beyond climate are important for improving the accuracy of habitat suitability models explaining species distributions. We found that a large portion of explained deviance in shorebird occupancy is related to direct effects of additional predictors, such as elevation and land cover, and that these effects are not well captured as indirect effects of climate. It is therefore unwise to assume that climate models are always sufficient for explaining habitat suitability.

By making these assumptions, we place unnecessary limits on our understanding of species relationships with their environment. As many countries seek to expand their protected areas networks to meet international commitments under the Convention on Biological Diversity [50], a more complete understanding of species' distributions can have important management implications now and especially in the future, under scenarios of climatic change.

Data Availability

All data and code files are available from the Zenodo database (https://doi.org/10.5281/zenodo.7613304).

Acknowledgements

This work was funded by Environment and Climate Change Canada, Arcticnet, NSERC, OGS, and the W Garfield Weston Foundation. We thank the field crews who conducted shorebird surveys for the PRISM program, and the Polar Continental Shelf program for providing logistical support.

References

1. Peterson AT, Soberón J. Species Distribution Modeling and Ecological Niche Modeling: Getting the Concepts Right. Nat Conserv. 2012;10(2):102–7.

2. Ramirez-Villegas J, Cuesta F, Devenish C, Peralvo M, Jarvis A, Arnillas CA. Using species distributions models for designing conservation strategies of Tropical Andean biodiversity under climate change. J Nat Conserv. 2014 Oct;22(5):391–404.

3. Fong A, Viña Dávila N, López-Iborra GM. Amphibian Hotspots and Conservation Priorities in Eastern Cuba Identified by Species Distribution Modeling. Biotropica. 2015 Jan;47(1):119–27.

4. Zhang MG, Zhou ZK, Chen WY, Slik JWF, Cannon CH, Raes N. Using species distribution modeling to improve conservation and land use planning of Yunnan, China. Biol Conserv. 2012 Sep;153:257–64.

5. Kramer AM, Annis G, Wittmann ME, Chadderton WL, Rutherford ES, Lodge DM, et al. Suitability of Laurentian Great Lakes for invasive species based on global species distribution models and local habitat. Ecosphere [Internet]. 2017 Jul [cited 2022 Oct 7];8(7). Available from: https://onlinelibrary.wiley.com/doi/10.1002/ecs2.1883

6. Root T. Environmental Factors Associated with Avian Distributional Boundaries. J Biogeogr. 1988 May;15(3):489.

7. Soberón JM. Niche and area of distribution modeling: a population ecology perspective. Ecography. 2010 Feb;33(1):159–67.

8. Langham GM, Schuetz JG, Distler T, Soykan CU, Wilsey C. Conservation Status of North American Birds in the Face of Future Climate Change. LaDeau SL, editor. PLOS ONE. 2015 Sep 2;10(9):e0135350.

9. Oliver TH, Morecroft MD. Interactions between climate change and land use change on biodiversity: attribution problems, risks, and opportunities. WIREs Clim Change. 2014 May;5(3):317–35.

10. Pearson RG, Dawson TP. Predicting the impacts of climate change on the distribution of species: are bioclimate envelope models useful? Glob Ecol. 2003;11.

11. Dyderski MK, Paź S, Frelich LE, Jagodziński AM. How much does climate change threaten European forest tree species distributions? Glob Change Biol. 2018 Mar;24(3):1150–63.

12. Graham EM, Reside AE, Atkinson I, Baird D, Hodgson L, James CS, et al. Climate change and biodiversity in Australia: a systematic modelling approach to nationwide species distributions. Australas J Environ Manag. 2019 Apr 3;26(2):112–23.

13. Elith J, Leathwick JR. Species Distribution Models: Ecological Explanation and Prediction Across Space and Time. Annu Rev Ecol Evol Syst. 2009 Dec 1;40(1):677–97.

14. de Araújo CB, Marcondes-Machado LO, Costa GC. The importance of biotic interactions in species distribution models: a test of the Eltonian noise hypothesis using parrots. Silman M, editor. J Biogeogr. 2014 Mar;41(3):513–23.

15. Jenkins DA, Lecomte N, Andrews G, Yannic G, Schaefer JA. Biotic interactions govern the distribution of coexisting ungulates in the Arctic Archipelago – A case for conservation planning. Glob Ecol Conserv. 2020 Dec;24:e01239.

16. Regos A, Gagne L, Alcaraz-Segura D, Honrado JP, Domínguez J. Effects of species traits and environmental predictors on performance and transferability of ecological niche models. Sci Rep. 2019 Dec;9(1):4221.

17. Wisz MS, Pottier J, Kissling WD, Pellissier L, Lenoir J, Damgaard CF, et al. The role of biotic interactions in shaping distributions and realised assemblages of species: implications for species distribution modelling. Biol Rev. 2013 Feb;88(1):15–30.

18. Sexton JP, McIntyre PJ, Angert AL, Rice KJ. Evolution and Ecology of Species Range Limits. Annu Rev Ecol Evol Syst. 2009 Dec 1;40(1):415–36.

19. Piedallu C, Gégout JC, Perez V, Lebourgeois F. Soil water balance performs better than climatic water variables in tree species distribution modelling: Soil water balance improves tree species distribution models. Glob Ecol Biogeogr. 2013 Apr;22(4):470–82.

20. Mod HK, Scherrer D, Luoto M, Guisan A. What we use is not what we know: environmental predictors in plant distribution models. Scheiner S, editor. J Veg Sci. 2016 Nov;27(6):1308–22.

21. Cunningham JA, Kesler DC, Lanctot RB. Habitat and social factors influence nest-site selection in Arctic-breeding shorebirds. The Auk. 2016 Jul;133(3):364–77.

22. Smith PA, Gilchrist HG, Smith JNM. Effects of nest habitat, food, and parental behaviour on shorebird nest success. The Condor. 2007;109:15–31.

23. Swift RJ, Rodewald AD, Senner NR. Breeding habitat of a declining shorebird in a changing environment. Polar Biol. 2017 Sep;40(9):1777–86.

24. Wauchope HS, Shaw JD, Varpe Ø, Lappo EG, Boertmann D, Lanctot RB, et al. Rapid climate-driven loss of breeding habitat for Arctic migratory birds. Glob Change Biol. 2017 Mar;23(3):1085–94.

25. Lathrop RG, Sacatelli R, Niles L, Mizrahi D, Smith J, Dey A. Arctic Shorebird Habitat Climate Change Resilience Analysis for the Arctic Migratory Birds Initiative (AMBI) - the Americas Flyway Action Plan. Montreal, Canada: Comission for Environmental Cooperation; 2019 p. 137.

26. Lathrop RG, Niles L, Smith P, Peck M, Dey A, Sacatelli R, et al. Mapping and modeling the breeding habitat of the Western Atlantic Red Knot (*Calidris canutus rufa*) at local and regional scales. The Condor. 2018 Aug;120(3):650–65.

27. Saalfeld ST, Lanctot RB, Brown SC, Saalfeld DT, Johnson JA, Andres BA, et al. Predicting breeding shorebird distributions on the Arctic Coastal Plain of Alaska. Ecosphere. 2013 Jan;4(1):art16.

28. R Core Team. R: A language and environment for statistical computing [Internet]. Vienna, Austria: R Foundation for Statistical Computing; 2022. Available from: https://www.R-project.org/

29. Bart JR, Johnston VH, editors. Arctic shorebirds in North America: a decade of monitoring. Berkley: Univ of California Press.; 2012. (Studies in avian biology; vol. 44).

30. Walker DA, Gould WA, Maier HA, Raynolds MK. The Circumpolar Arctic Vegetation Map: AVHRR-derived base maps, environmental controls, and integrated mapping procedures. Int J Remote Sens. 2002 Jan;23(21):4551–70.

31. Fick SE, Hijmans RJ. WorldClim 2: new 1-km spatial resolution climate surfaces for global land areas. Int J Climatol. 2017 Oct;37(12):4302–15.

32. Patterson T, Vaughn Kelso N. Natural Earth [Internet]. 2022 [cited 2022 Aug 18]. Available from: naturalearthdata.com

33. O'Leary III D, Hall DK, Medler M, Matthews R, Flower A. Snowmelt Timing Maps Derived from MODIS for North America, Version 2, 2001-2018 [Internet]. ORNL DAAC, Oak Ridge, Tennessee, USA; 2019. Available from: https://doi.org/10.3334/ORNLDAAC/1712

34. Olthof I, Latifovic R, Pouliot D. Development of a circa 2000 land cover map of northern Canada at 30 m resolution from Landsat. Can J Remote Sens. 2009;35(2):14.

35. Jackson HB, Fahrig L. What size is a biologically relevant landscape? Landsc Ecol. 2012 Aug;27(7):929–41.

36. McGarigal K, Wan HY, Zeller KA, Timm BC, Cushman SA. Multi-scale habitat selection modeling: a review and outlook. Landsc Ecol. 2016 Aug;31(6):1161–75.

37. Lefcheck JS. piecewiseSEM: Piecewise structural equation modeling in R for ecology, ecolution, and systematics. Methods Ecol Evol. 2016;7(5):573–9.

38. Murphy M. semEff: Automatic Calculation of Effects for Piecewise Structural Equation Models [Internet]. 2022. Available from: https://CRAN.R-project.org/package=semEff

39. Keith TZ. Multiple regression and beyond: an introduction to multiple regression and structural equation modeling. 2nd edition. New York ; London: Routledge, Taylor & Francis Group; 2015. 592 p.

40. Walker DA. Hierarchical subdivision of Arctic tundra based on vegetation response to climate, parent material and topography. Glob Change Biol. 2000 Dec;6(S1):19–34.

41. Pöyry J, Luoto M, Heikkinen RK, Saarinen K. Species traits are associated with the quality of bioclimatic models. Glob Ecol Biogeogr. 2008 May;17(3):403–14.

42. Kadmon R, Farber O, Danin A. A systematic analysis of factors affecting the performance of climatic envelope models. Ecol Appl. 2003 Jun;13(3):853–67.

43. Wiens JA. Spatial Scaling in Ecology. Funct Ecol. 1989;3(4):385.

44. Mackey BG, Lindenmayer DB. Towards a hierarchical framework for modelling the spatial distribution of animals: Modelling the spatial distribution of animals. J Biogeogr. 2001 Dec 21;28(9):1147–66.

45. Luoto M, Virkkala R, Heikkinen RK. The role of land cover in bioclimatic models depends on spatial resolution. Glob Ecol Biogeogr. 2006 Aug 29;0(0):061120101210017-???

46. Bucklin DN, Basille M, Benscoter AM, Brandt LA, Mazzotti FJ, Romañach SS, et al. Comparing species distribution models constructed with different subsets of environmental predictors. Thuiller W, editor. Divers Distrib. 2015 Jan;21(1):23–35.

47. Dubuis A, Giovanettina S, Pellissier L, Pottier J, Vittoz P, Guisan A. Improving the prediction of plant species distribution and community composition by adding edaphic to topo-climatic variables. Rocchini D, editor. J Veg Sci. 2013 Jul;24(4):593–606.

48. Titeux N, Henle K, Mihoub JB, Regos A, Geijzendorffer IR, Cramer W, et al. Biodiversity scenarios neglect future land-use changes. Glob Change Biol. 2016 Jul;22(7):2505–15.

49. Verburg PH, van Asselen S, van der Zanden EH, Stehfest E. The representation of landscapes in global scale assessments of environmental change. Landsc Ecol. 2013 Jul;28(6):1067–80.

50. Le Prestre PG, editor. Governing global biodiversity: The evolution and implementation of the convention on biological diversity. Routledge; 2017.

51. Léandri-Breton DJ, Bêty J. Vulnerability to predation may affect species distribution: plovers with broader arctic breeding range nest in safer habitat. Sci Rep. 2020 Dec;10(1):5032.

Supporting Information

S1 Fig. Maps of northern Canada showing where each species of breeding shorebirds was present during PRISM surveys 1994-2018.

S1 Table. Years in which each PRISM region was surveyed. See (29) for map of survey regions.

S2 Table. Range of values for environmental predictors.

S3 Table. Radius (m) of the optimal scale of effect at which land cover affects shorebird occupancy, determined by AIC.

S4 Table. Full results of GLMs used for deviance partitioning.

S5 Table. Path analysis: summary of the significant total effects of predictors on shorebird occupancy.

S6 Table. Path analysis: full results of the 17 species-specific path models, including summaries of the direct, indirect and total effects of the paths between all variables.

Figure 1

Click here to access/download;Figure;Figure 1.tif

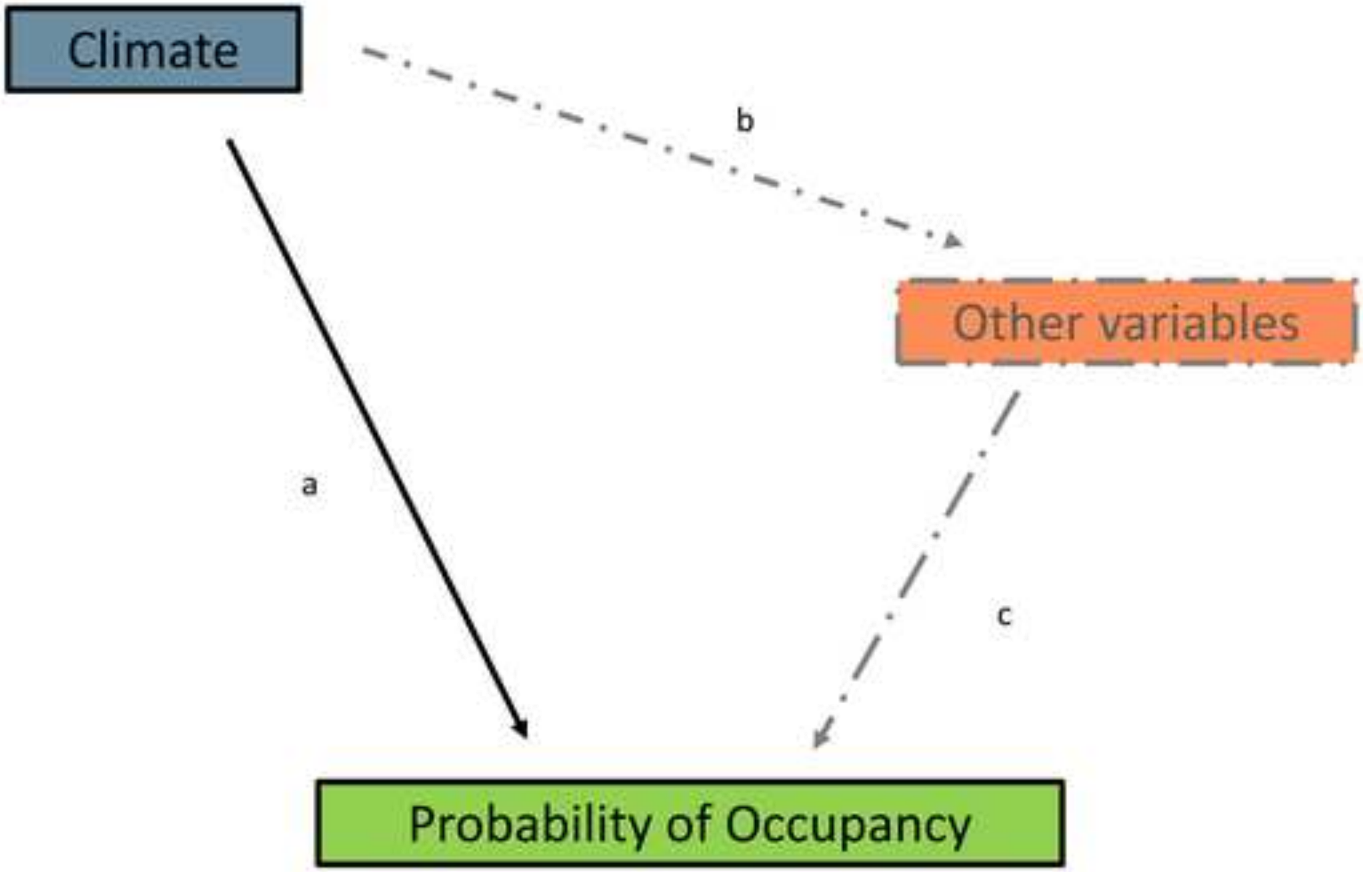

Figure 2

Click here to access/download;Figure;Figure 2.tif

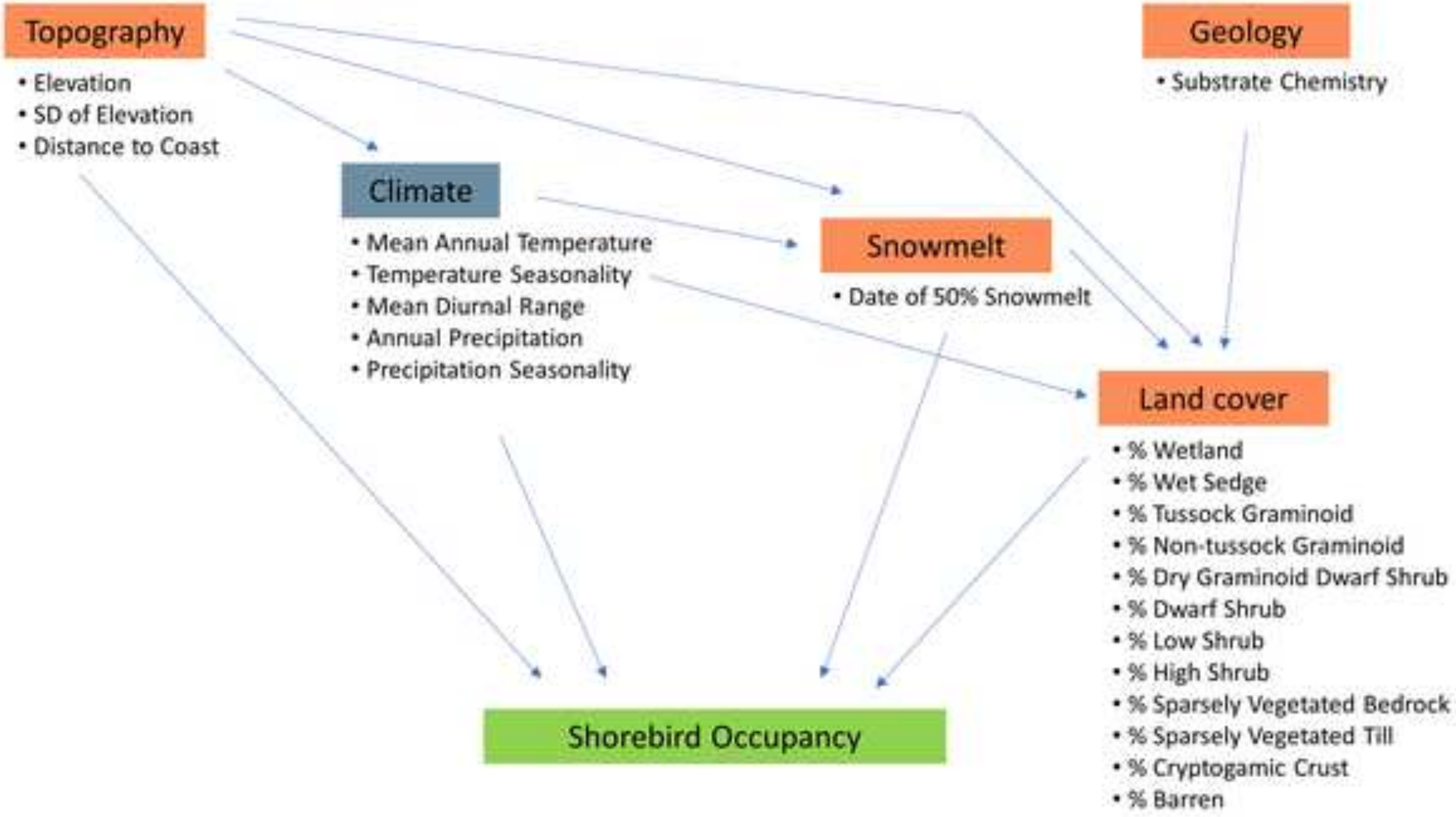

Figure 3

Click here to access/download;Figure;Figure 3.tif

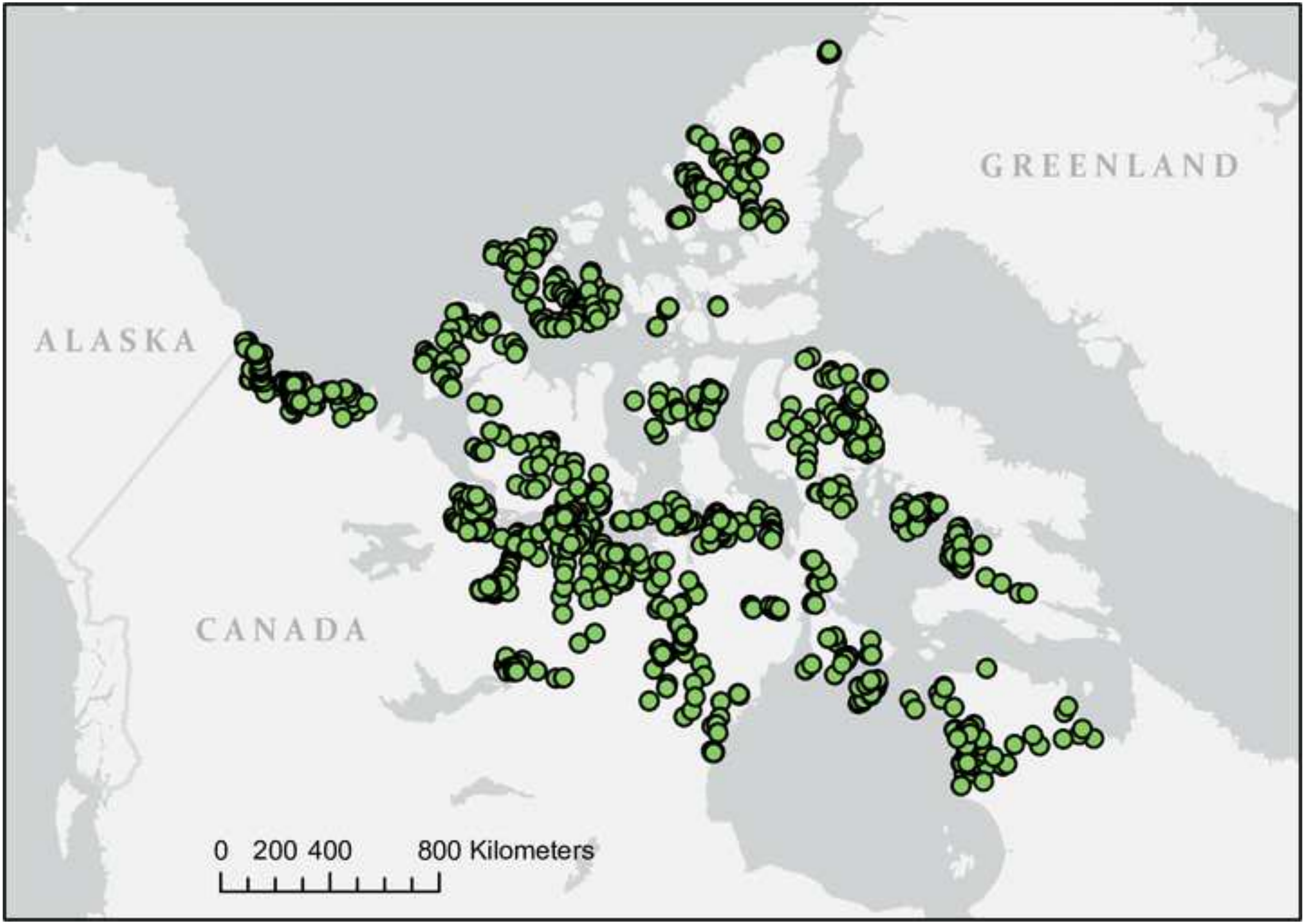

Figure 4

Click here to access/download;Figure;Figure 4.tif

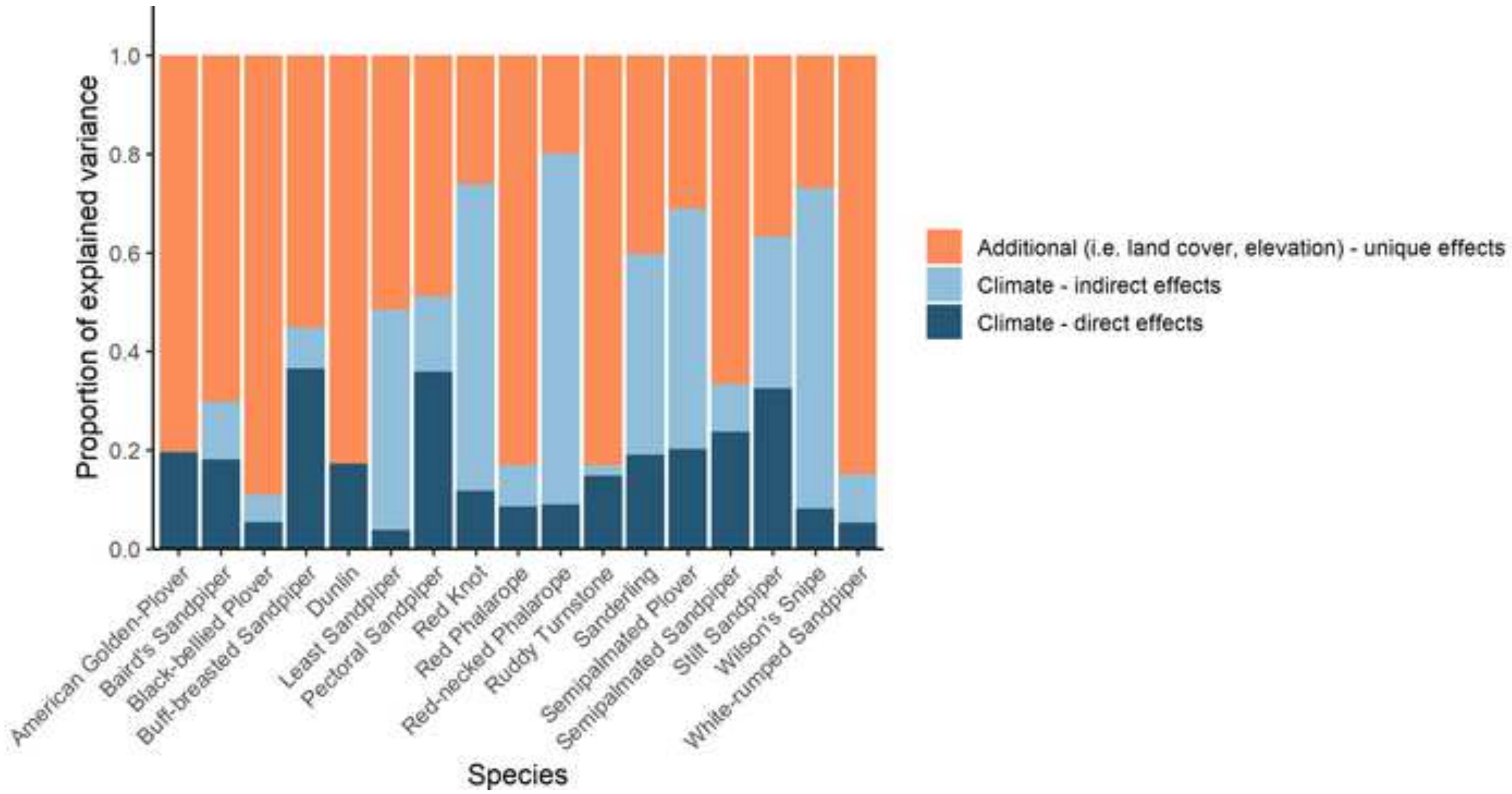

Figure 5

Click here to access/download;Figure;Figure 5.tif

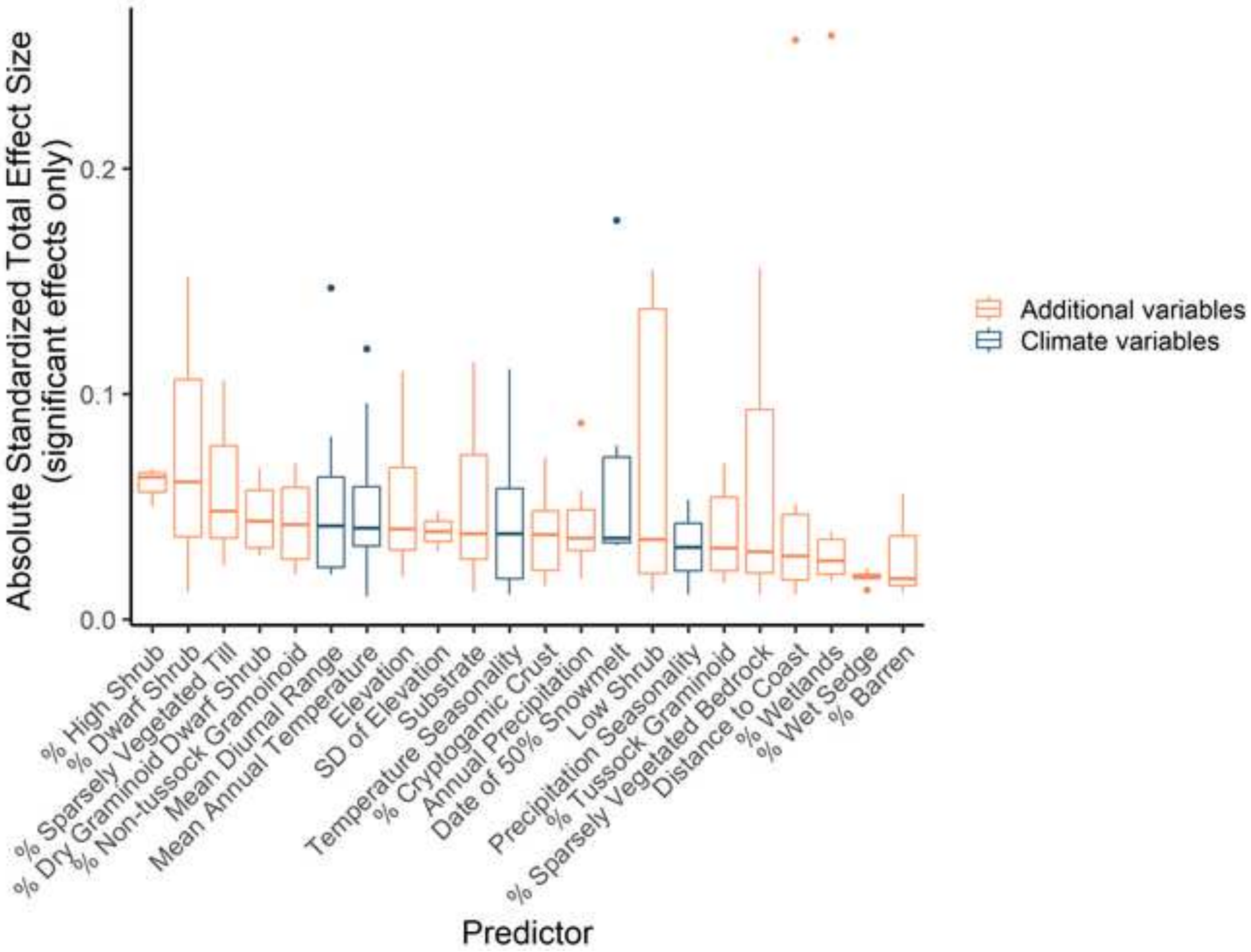

Click here to access/download
Supporting Information
S1 Fig.pdf

Click here to access/download
Supporting Information
S1 Table.docx

Click here to access/download
Supporting Information
S2 Table.docx

Click here to access/download
Supporting Information
S3 Table.docx

Click here to access/download
Supporting Information
S4 Table.txt

Click here to access/download
Supporting Information
S5 Table.csv

Click here to access/download
Supporting Information
S6 Table.csv
